# Governing evolution: A socioecological comparison of resistance management for insecticidal transgenic Bt crops among four countries

**DOI:** 10.1007/s13280-019-01167-0

**Published:** 2019-03-21

**Authors:** Yves Carrière, Zachary S. Brown, Sharon J. Downes, Govind Gujar, Graham Epstein, Celso Omoto, Nicholas P. Storer, David Mota-Sanchez, Peter Søgaard Jørgensen, Scott P. Carroll

**Affiliations:** 1 Department of Entomology, University of Arizona, Tucson, USA; 2 Department of Agricultural and Resource Economics, North Carolina State University, Raleigh, USA; 3 CSIRO Agriculture and Food, Narrabri, Australia; 4 South Asia Biotechnology Centre, New Delhi, India; 5 School of Environment, Resources and Sustainability, University of Waterloo, Waterloo, Canada; 6 Department of Entomology and Acarology, University of Sao Paulo, Sao Paulo, Brazil; 7 Corteva AgriscienceTM, Agriculture Division of DowDuPont, Wilmington, USA; 8 Department of Entomology, Michigan State University, East Lansing, USA; 9 Global Economic Dynamics and the Biosphere, Royal Swedish Academy of Sciences, Lilla Frescativägen 4a, 10405 Stockholm, Sweden; 10 Stockholm Resilience Centre, Stockholm University, Kräftriket 2B, 10691 Stockholm, Sweden; 11 Department of Entomology and Nematology, University of California at Davis, Davis, USA

**Keywords:** *Bacillus thuringiensis* crops, Conservation and monitoring, Institutional analysis and development, Sustainability, Transgenic crops

## Abstract

Cooperative management of pest susceptibility to transgenic *Bacillus thuringiensis* (Bt) crops is pursued worldwide in a variety of forms and to varying degrees of success depending on context. We examine this context using a comparative socioecological analysis of resistance management in Australia, Brazil, India, and the United States. We find that a shared understanding of resistance risks among government regulators, growers, and other actors is critical for effective governance. Furthermore, monitoring of grower compliance with resistance management requirements, surveillance of resistance, and mechanisms to support rapid implementation of remedial actions are essential to achieve desirable outcomes. Mandated resistance management measures, strong coordination between actors, and direct linkages between the group that appraises resistance risks and growers also appear to enhance prospects for effective governance. Our analysis highlights factors that could improve current governance systems and inform other initiatives to conserve susceptibility as a contribution to the cause of public good.

**Electronic supplementary material:**

The online version of this article (10.1007/s13280-019-01167-0) contains supplementary material, which is available to authorized users.

## Introduction

Pesticides and antimicrobials enhance food security and human health by controlling pests and pathogens, but can also contribute to increased vulnerability by reducing the diversity of control methods and increasing dependence on new technologies. Complementary alternatives for managing the evolution of resistance in targeted organisms are underdeveloped and fraught with challenges (Living with Resistance Project 2018) . Consequently, there is an urgent need to better understand the diversity and relative efficacy of practices for sustaining biocide susceptibility (Carroll et al. 2014). In this paper, we draw upon the efforts of four countries to govern the management of insect pest resistance to transgenic *Bacillus thuringiensis* (Bt) crops, using a socioecological system approach to highlight the diversity of contexts in which biocide susceptibility problems and the design of contextually explicit solutions emerge.

The widespread use of Bt crops to manage insect pests, especially in corn, cotton, and soybeans, has transformed agriculture in several countries over the last two decades (James 2017). These crops benefit farmers by protecting yield from damage through insect feeding, simplifying pest management, and reducing reliance on synthetic insecticides. These crops also contribute to the cause of public good by reducing crop losses, stabilizing yield, reducing regional pest abundance and use of insecticide sprays, and improving integrated pest management (IPM) (Naranjo 2010; Klümper and Qaim 2014; NASEM 2016; Baker and Tann 2017; Downes et al. 2017; Dively et al. 2018). However, many economically important pests have evolved resistance to Bt crops, which threatens these benefits (Tabashnik and Carrière 2017).

Pest susceptibility to Bt crops is a common-pool resource (CPR) in which the private benefits of using Bt crops contribute to overuse of the resource (Vacher et al. 2006). By adopting practices that favor local evolution and subsequent spread of pest resistance, one grower using Bt seed can contribute to depleting the stock of Bt susceptibility available to all growers (Carrière et al. 2010; Andow et al. 2016). Because of this, several countries have developed governance systems to help preserve the public benefits that the use of Bt crops provides (Downes and Mahon 2012; Matten et al. 2012).

Standard prescriptions for efficient management of CPRs involve policies that provide incentives for their conservation (Clark 2010). In the case of Bt crops, policies to slow evolution of pest resistance have primarily mandated that growers plant a portion of their land with a non-Bt “refuge” to breed susceptible pests as a means of slowing resistance evolution (Section “The refuge strategy”). While this approach yields long-term benefits (Qiao et al. 2008), growers have private incentives to maximize short-term profits by reducing the size and quality of refuges (Section “Institutional analysis for Bt resistance governance”). In response, many countries attempt to improve stewardship of Bt susceptibility through regulations and support of collaborations among diverse participants. However, outcomes vary widely and are influenced by a range of factors (Section “Linkage among participants in different countries and opportunities for resistance management”). We therefore adapt the Institutional Analysis and Development (IAD) framework (Ostrom 2005) to better understand the different approaches to resistance management in Australia, Brazil, India, and the USA, and generate insights into the types of policies and strategies that are more (or less) likely to drive successful outcomes in different contexts. These countries were chosen because of their extensive use of Bt crops and differences in the ecological and socioeconomic attributes of Bt resistance management.

## The refuge strategy

Planting refuges of non-Bt crops has been the prominent tactic to delay resistance evolution to Bt crops, although adoption of other IPM measures such as crop rotation is required in some contexts (Andow et al. 2016; Carrière et al. 2016; Appendix S1). In the following sections, we refer to insecticide resistance management (IRM) as the use of refuges and other IPM tactics to manage resistance evolution to Bt crops.

In this context, we overview the biological basis for using refuges. For simplicity, we consider a refuge strategy for single-toxin Bt crops (e.g., crop producing Cry1Ac), which were the first commercialized. Refuges are also needed to delay resistance to pyramided Bt crops, which produce two or more toxins (e.g., Cry1Ac and Cry2Ab) targeting the same pest and are more effective than single-toxin crops for delaying resistance (Carrière et al. 2016). For single-toxin Bt crops, refuges work best if the alleles conferring resistance are recessive (see Table 1 for definitions of biological terms). Before crops with novel Bt toxins are introduced, such resistance alleles are typically rare, and homozygous-resistant individuals, even more so. If refuges are effective, a relatively high number of Bt-susceptible individuals move from them to Bt crops and mate with the rare homozygous-resistant surviving individuals. The progeny from these matings carry only one resistance allele and are killed by Bt plants (because resistance is recessive), thereby delaying resistance evolution (Carrière et al. 2010).

**Table 1:** Genetic factors affecting resistance evolution. Adapted from Tabashnik et al. (2014)

General terms
Allele: any of the many forms of a gene
Fitness: the ability to survive and produce offspring relative to individuals of the same species
Homozygous individuals: Individuals that carry two copies of the same allele
Recessive resistance: inheritance of resistance in which only individuals with two resistance alleles at the locus that controls susceptibility show a decrease in susceptibility to a Bt toxin
Genetic factors
Cross-resistance: resistance to a Bt toxin caused by evolution of resistance to a different toxin
Fitness cost: a tradeoff in which resistance alleles increase fitness in environments with a Bt toxin but reduce fitness in environments without the toxin
Incomplete resistance: resistance in which fitness is lower for resistant individuals exposed to a Bt toxin relative to resistant individuals not exposed to the toxin
Inheritance of resistance: the susceptibility to a Bt toxin of individuals with one resistance allele and one susceptibility allele at the locus that controls susceptibility, relative to the susceptibility of individuals with two susceptibility alleles and individuals with two resistance alleles
Practical resistance: field-evolved resistance of sufficient magnitude to reduce the efficacy of a Bt crop against a pest
Redundant killing: each toxin alone in a pyramided Bt crop kills nearly all individuals with two susceptibility alleles at the locus that controls susceptibility

## Institutional analysis for Bt resistance governance

**Fig. 1:**
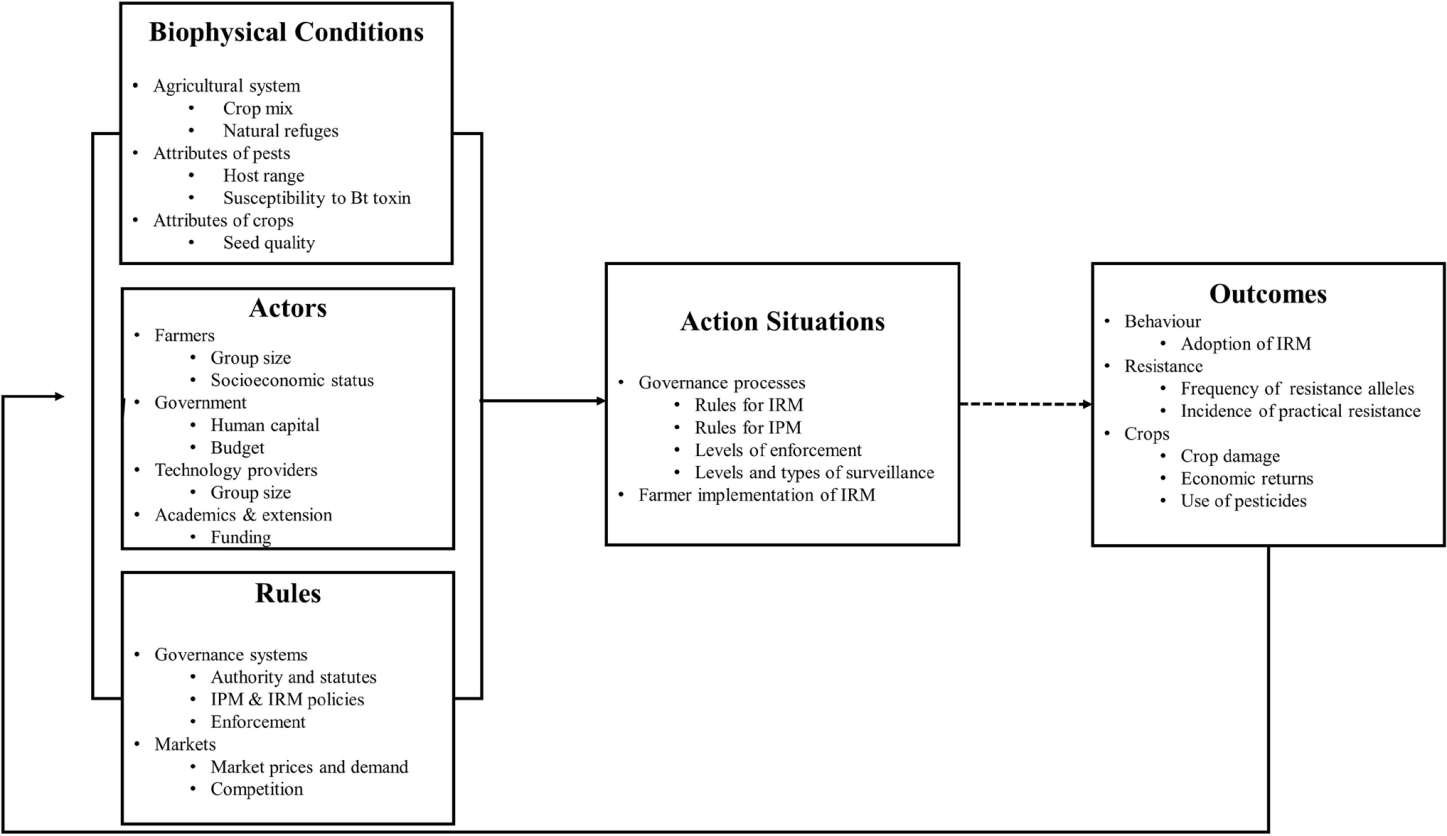
Overview of the Institutional Analysis and Development framework for governance of Bt crops. The bulleted list of factors is not comprehensive. See Sections “Biophysical conditions”, “Outcomes”, “Rules”, and “Actors and action situations” for details on factors that influence resistance outcomes

The IAD framework adapted for Bt resistance governance highlights how attributes of the environment, actors, and rules interact to influence the development and performance of policies (Fig. 1). This framework supports a detailed analysis of the influence of ecological and socioeconomic factors on incentives and resistance outcomes. Our application frames the system as a society–biology loop (Spangenberg et al. 2015), in which adoption of IRM by growers influences the evolution of resistance and vulnerability to pest damage (Fig. 1, broken arrow), which trigger changes in incentives for resistance management (Fig. 1, path defined by continuous arrows). In the following subsections, we outline the main factors that influence resistance outcomes (Fig. 1). Using the four country case studies, we then describe (Section “Linkage among participants in different countries”) and analyze (Section “Opportunities for resistance management”) the dynamics of interactions embedded within this loop.

### Biophysical conditions

Biophysical conditions that affect resistance evolution are grouped into three broad classes: (1) pest biology and ecology, (2) genetic factors, and (3) characteristics of Bt crops and agricultural landscapes.

#### Attributes of pests

The number of host plant species exploited by pests must be considered for managing resistance. For specialized pests, planting specific refuges of non-Bt crops is essential. Planted refuges may be less important for pests that feed on many non-Bt host plant species (i.e., polyphagous pests). For example, refuges of non-Bt cotton were discontinued in most of the USA Cotton Belt when studies indicated that non-cotton plants provide many wild-type moths of the key target pests in Bt cotton fields of these regions (EPA 2007). Similarly in India, pigeon pea planted on marginal lands for food security may act as a refuge for the cotton bollworm, *Helicoverpa armigera* (Karihaloo and Kumar 2009). The efficacy of alternative refuge plants varies among pests and regions and must be evaluated on a case-by-case basis (Brévault et al. 2012; van den Berg 2017).

The several genetic factors affecting the evolution of resistance are listed in Table 1. Other relevant factors include the number of generations per year or season, mating behavior, and mobility, all of which potentially influencing host use and movement between refuges and Bt crops (Fitt et al. 2004; Matten et al. 2012). Simulation models are a primary tool to integrate impacts of these factors for development of IRM tailored to a particular pest and region (Matten et al. 2012). Ultimately, implementation of IRM must consider all key pests and Bt crops in a region.

#### Attributes of agricultural landscapes and crops

The presence of similar Bt toxins in different host crop types can increase refuge needs for polyphagous pests because such crops select for resistance concurrently instead of independently (Carrière et al. 2016). This occurs in parts of the USA Cotton Belt where *Helicoverpa zea* exploits several types of Bt corn and cotton, and in the Cerrado region of Brazil where the fall armyworm and *Helicoverpa spp.* feed on several types of Bt corn, cotton, and soybean (Appendix S2). By contrast, only one type of Bt crop (cotton) is used in Australia (Appendix S1).

Similarly, concurrent use of single-toxin and pyramided crops with similar toxins can accelerate the evolution of resistance to single-toxin crops and reduce the durability of pyramided crops (e.g., Brazil and the USA) (Carrière et al. 2016). This risk is present in India where commercial Cry1Ac cotton was officially replaced by Cry1Ac + Cry2Ab cotton (Desai et al. 2016) but persists in unapproved forms (see below). By contrast, full replacement of single-toxin by two-toxin cotton, and two-toxin cotton by three-toxin cotton, was achieved in one year in Australia (Appendix S1). Similarly, the IRM value of a pyramided crop is reduced when it is introduced after evolution of pest resistance to a related single-toxin crop (Carrière et al. 2016; Tabashnik and Carrière 2017).

Areas dominated by a few large farms can facilitate cooperative efforts in areawide pest control (Singerman et al. 2017), which can include resistance management. In contrast, a major constraint to adopting IPM in developing countries is the concern that pest problems transcend the borders of growers’ fields (Parsa et al. 2014). In regions with many small farms, this concern could undermine adoption of IRM. Research and extension (e.g., demonstration farms) focused on this issue could facilitate cooperative resistance management (Llewellyn and Allen 2006).

Resistance evolution is affected by grower cultivation practices that influence, or are influenced, by landscape characteristics. For example, rotation of Bt corn with other crops increases crop diversity, yield, and biocontrol services (Robertson et al. 2014), and also delays resistance evolution to Bt corn in corn rootworm (Andow et al. 2016). Insecticide sprays that kill susceptible insects in refuges of non-Bt crops may accelerate the evolution of resistance (Matten et al. 2012). Effectiveness of non-Bt crop refuges is thus linked to the need for chemical control of the local pest complexes and grower tolerance for yield reduction.

Evolution of resistance is influenced by characteristics of Bt and non-Bt crops. Cry1Ac cotton was officially introduced in India in 2002 and resistance in pink bollworm, *Pectinophora gossypiella*, was first observed in 2009 in Gujarat (Dhurua and Gujar 2011). However, unapproved Bt cotton seed was sold before 2002, and many unauthorized varieties of Cry1Ac cotton were available between 2003 and 2008 (Lalitha et al. 2009). Only approved Bt cotton seed was sold with refuge seed. Accordingly, tolerance of unauthorized Bt cotton by the government contributed to limited use of refuges (Lalitha et al. 2009) and rapid evolution of pink bollworm resistance to Cry1Ac cotton (Mohan et al. 2016). In China, Cry1Ac cotton was introduced in 2000 but refuges are not mandated despite early warnings of resistance evolution (Liu et al. 2017). Starting in 2009, growers considerably increased their use of F_2_ seed, produced by self-pollination of F_1_ Bt cotton hybrids, because this strategy lowered costs for insecticides and seed (Wan et al. 2017). F_2_ seed yield 25% non-Bt plants, which increased the area of refuge within Bt crop fields and led to declines in pink bollworm resistance (Wan et al. 2017).

### Outcomes

The most direct outcome for evaluating Bt resistance management is the level of resistance in target pest populations (Tabashnik et al. 2014). Small increases in frequencies of resistance alleles do not necessarily result in crop losses. Practical resistance occurs when frequencies of resistance alleles are high enough to have negative effects for pest control (Tabashnik et al. 2014). Key outcomes related to practical resistance include reduced crop yields, increases in pesticide use, and associated changes in revenues and production costs. Because the risk of rapid resistance evolution is mitigated by proactive IRM, measuring adoption of IRM tactics is important for mediating resistance outcomes (Jaffe 2009; Reisig 2017). Early detection of increased frequencies can warn that changes in IRM may be needed to delay practical resistance (Downes et al. 2010; Liu et al. 2017; Appendix S1).

The primary economic objective of resistance management is to maximize the value of current pest control technology over time, recognizing that greater use of Bt crops generally depletes pest susceptibility more rapidly (Hueth and Regev 1974; Appendix S5). This objective involves considering the current and future benefits of the technology relative to alternative economic investments. For example, increasing refuge areas may reduce current yields. Strategic alternatives would thus use the proceeds resulting from not increasing refuge areas to deploy alternative pest control tactics to sustain yields (Laxminarayan and Simpson 2002). In the case of Bt crops, such investments could include new insecticidal gene products or different pest control tactics such as biocontrol. However, the future availability and success of novel control measures, including insecticidal technology such as RNAi, is uncertain (Tabashnik and Carrière 2017).

Other economic evaluation criteria span grower and technology providers’ profits and consumer benefits (NRC 2010). Economic criteria can be further expanded to include the monetized value of environmental impacts such as the reduction of insecticide sprays (NASEM 2016). Beyond economic evaluation, broader socioecological criteria include equity concerns (e.g., should reductions in food prices receive more weight than economic benefits for growers, rural economies or biotech companies?) and resilience of food systems (Ericksen 2008).

### Rules

Formal and informal institutions, including policies, rules, and norms, influence resistance by affecting incentives and constraints on participants’ actions. Australia, India and the USA formally mandate IRM and reporting of pest resistance, although enforcement and penalties for noncompliance vary (Section “Linkage among participants in different countries”). Technology providers can be directly (e.g., USA) or indirectly (e.g., Australia) responsible for enforcing IRM mandates, or can voluntarily coordinate efforts (e.g., Brazil) to improve grower implementation (Section “Linkage among participants in different countries”). Grower associations also provide incentives for IRM, as exemplified by the *my*BMP program in Australia (Appendix S1). In some cases, public research and extension services have built trust with growers through long-term relationships, helping to support planting of refuges (Carrière et al. 2001; Downes et al. 2017). Social marketing campaigns by the private sector that appeal to stewardship norms may also influence growers’ resistance management practices (Brown 2018).

Resistance management is affected by broader institutional settings, including agricultural markets and government subsidies. Markets and economic forces influence incentives to deploy refuges, either intentionally or by affecting demand for Bt crops. Positive economic incentives for growers to plant Bt crops or refuges are potential yield gains, labor savings, and reduced pesticide expenditure, balanced against the price premium for Bt seeds (Useche et al. 2009). Crop prices also determine profit gains from planting Bt crops and implementing IRM. For example in the USA, higher corn prices resulting from expanding ethanol production increased use of Bt corn and reduced corn rotations with other crops, thereby neglecting an important IRM practice for Bt corn targeting corn rootworm (Fausti 2015). Agricultural subsidies are used extensively in many countries to stabilize the agricultural sector (Glauber 2013). Making grower eligibility for such subsidies conditional on practicing IRM could be a further means of stabilizing the sector (Section “Matching incentives to context”).

Intellectual property rights are another dimension of Bt resistance management rules. Technology providers derive profits from legal monopolies created by patents, allowing them to charge a premium for Bt seed. This generates an incentive to prioritize improvements in Bt seed to obtain the greatest returns on investment (Shi et al. 2013; Global AgriSystem 2016). In the USA, such practices likely contributed to reductions in the relative quality and availability of non-Bt seed (NRC 2010), which has deterred refuge planting (Reisig 2017).

The agricultural biotechnology sector has consolidated over the past decades and is now dominated by a few firms (Howard 2015), which has facilitated coordination on resistance management in the USA through the industry-led Agricultural Biotechnology Stewardship Technical Committee (ABSTC). However, principles of free-market competition appear to limit such coordination efforts. For example, the United States Environmental Protection Agency (EPA) requires each technology provider to maintain a list of customers found to be out of compliance with refuge requirements twice within a five-year period, and deny those growers access to their own Bt seed that require structured refuge (US EPA 2018). However, this only applies to the particular technology provider and does not prevent growers from purchasing Bt seed from a different provider.

### Actors and action situations

Participants in Bt resistance governance include growers, government regulators, non-governmental organizations, public research scientists, and extension agents, seed and input distributors, technology providers, and ultimately consumers. Subsets of these participants, each with their own objectives and constraints, interact in multiple-action situations to influence resistance outcomes.

Individual growers directly bear the near-term economic costs of refuge adoption but only derive longer-term benefits of refuges through preservation of Bt effectiveness. Accordingly, growers may perceive that they have little short-term incentive to plant refuges even if as a group they can gain from widespread adoption of the practice (Jaffe 2009; Reisig 2017). Importantly, awareness of the risk of resistance and compliance to IRM guidelines can be significantly enhanced when growers experience “resistance crises,” in which no pesticides remain effective to control pest populations (Ellsworth and Martinez-Carillo 2001; Downes et al. 2017; ISAA 2018).

Grower decisions to implement IRM are the core actions affecting Bt resistance evolution. It is often assumed that growers seek to maximize current profits. However, this model needs to be expanded to include additional motives related to the sustainability of Bt technology and grower preferences for non-Bt varieties (Birol et al. 2008). Additionally, growers could often value the welfare of neighboring growers and environmental quality of future generations due to altruistic motives, legacy concerns, or “enlightened self-interest” (Besser et al. 2004). Research on CPRs reveals that many users behave as conditional cooperators, being willing to preserve the resource conditional on their peers doing the same (Rustagi et al. 2010). Such motives raise opportunities for voluntary approaches to resistance governance by invoking shared norms about agricultural stewardship (Brown 2018). Grower attributes are also important, enabling or constraining cooperation among participants. Enablers generally include factors such as small group size, social cohesion, and cultural homogeneity among local grower groups (Section “Opportunities for resistance management”) as well as levels of socioeconomic development and access to capital (Baland and Platteau 1999; Habyarimana et al 2007).

Incentives for technology providers are shaped by intellectual property rights and market structure. Patent rights in most countries last up to 20 years, after which other companies may freely use the technologies. Thus, the degree to which technology providers have incentives to preserve effectiveness of a Bt crop might depend on the remaining patent life and replacement pest control technologies under development (Parisi et al. 2016). Technology provider investments in novel Bt crops and new modes of action have been critical to mitigate impacts of resistance to Bt crops (Tabashnik and Carrière 2017). Technology providers have increasingly marketed pyramided instead of single-toxin Bt crops, which enhances resistance management and allows reduced refuge areas (Downes and Mahon 2012; Carrière et al. 2016). Technology providers have also increased the availability of seed mixtures, which provide a random assortment of Bt and non-Bt plants side-by-side within fields (Carrière et al. 2016). Seed mixtures provide advantages for IRM relative to planting Bt crops and refuges in separate fields, such as ensuring widespread and uniform use of refuges and similar management of refuge and Bt plants. Seed mixtures also have disadvantages, such as facilitating evolution of resistance in pest species with larvae capable of moving among adjacent plants (Brévault et al. 2015; Carrière et al. 2016).

Government participants can be involved in many relevant action situations, including policy development, engagement, and appraisal of resistance risks. In the USA, EPA claimed regulatory authority for managing resistance to Bt crops. Historically, EPA has been granted regulatory authority for pesticides and Bt crops to increase the influence of environmental considerations on regulations and provide mechanisms to reconcile the divergent interests of growers, technology providers, and society (Sexton et al. 2007; Meghani and Kuzma 2011). In many other countries, agricultural authorities have primary jurisdiction for Bt resistance management.

## Linkage among participants in different countries

Here we describe relationships among actors influencing adoption and effectiveness of IRM in Australia, Brazil, India, and the USA. Determining linkages among participants is important for understanding governance outcomes, as such linkages influence the knowledge, opportunities, and constraints in play when actors are making decisions. Additional detailed information on resistance management for Bt crops in individual countries, including a side-by-side figure outlining linkages in the four countries, is provided in the Appendix.

### Australia (Fig. 2)

The Office of the Gene Technology Regulator (OGTR) assesses the risks of the technology to human and environmental health and safety and issues plans to mitigate them, which may include licensing conditions for the registrant (i.e., technology provider). To ensure appropriate resistance management, the Australian Pesticides and Veterinary Medicines Authority (APVMA) then works with the registrant and an industry expert committee (the Transgenic and Insect Management Strategies, TIMS). A peak industry body (Cotton Australia) facilitates TIMS’s work. The APVMA seeks endorsement from TIMS on required plans and considers variations submitted by the registrant. Growers sign contracts with the registrant, which mandate adherence to the plan. The registrant is responsible for auditing planting dates, refuges, and end of season destruction of pest populations, and reports compliance to TIMS and the APVMA. The registrant reserves the right to prevent future use of their product by growers who do not comply.

**Fig. 2:**
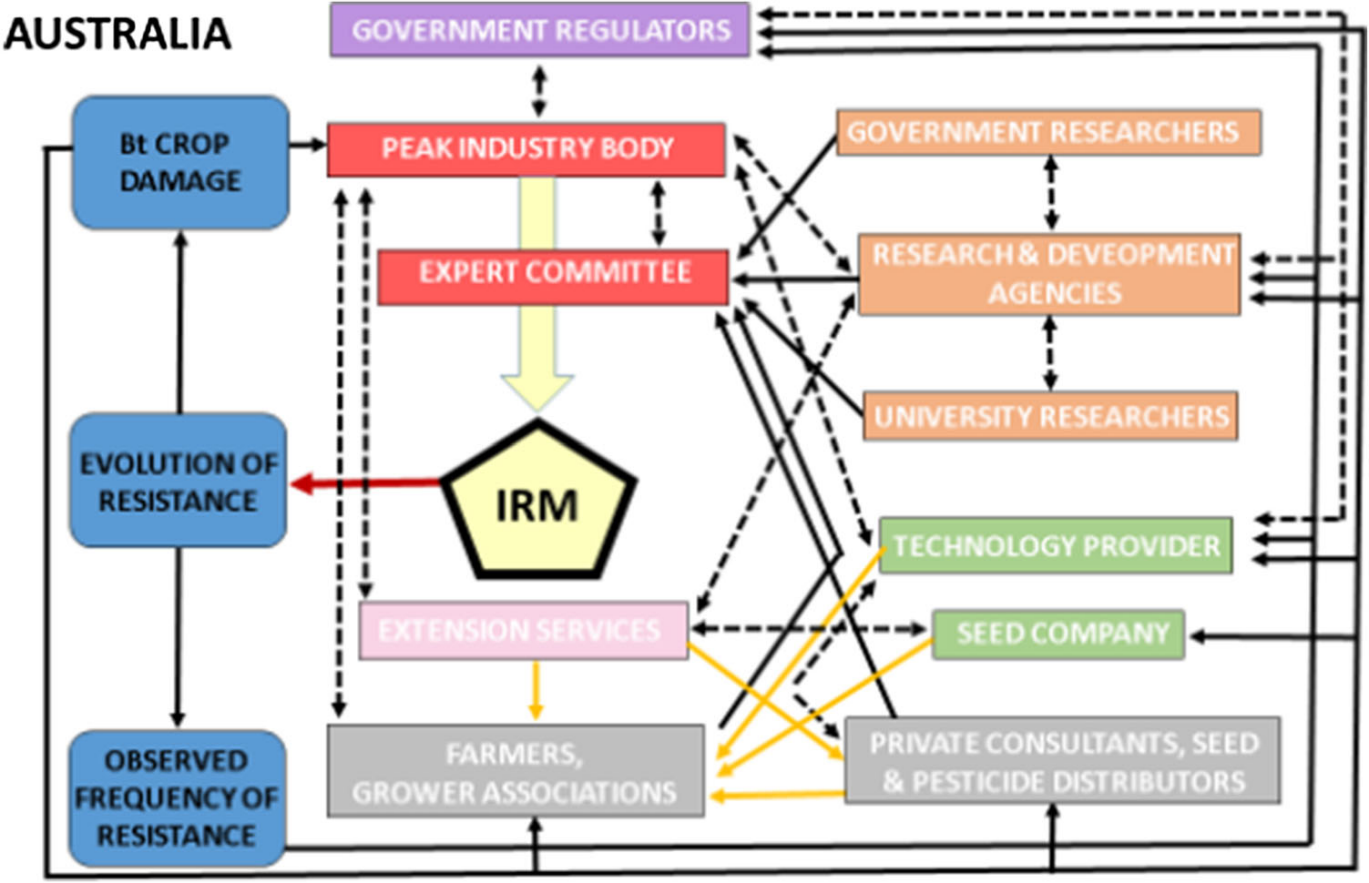
Influence diagram for Bt resistance management in Australia. Governance in Australia is characterized by many linkages, a strong top-down approach facilitated by a peak industry body, and a streamlined system (single technology provider). Red arrow indicates a reduction in evolution of resistance following implementation of IRM. Yellow arrows indicate stakeholders that directly promote implementation of IRM by growers. Black arrows indicate the influence of a stakeholder on actions taken by other stakeholders, of a variable on another variable, or of a variable on actions taken by stakeholders (without implying whether this influence is positive or negative). Double-pointed dashed black arrows indicate mutual influence between stakeholders. The larger yellow arrow indicates significant influence of a stakeholder group on IRM. A thick border around the IRM shape indicates an audited mandatory strategy. For simplicity, we grouped “Farmers and Grower Associations” and “Private consultants, seed and pesticide distributors” within a single category, although in reality the individual entities do not necessarily always operate in the same fashion with respect to IRM governance

Before registration, OGTR considers information from the registrant regarding (1) biology of the parent organism, (2) method and nature of the genetic modification and effect of introduced genes, (3) characteristics of the receiving environment, and (4) relevant Australian and international approvals. Post-registration, the registrant is responsible for working with TIMS to annually provide evidence to APVMA that resistance is being effectively managed including (1) user awareness of, and compliance with the Resistance Management Plan (RMP, equivalent to IRM in Fig. 2); (2) effectiveness of the RMP including monitoring for changes in resistance to Bt toxins in target pests; and (3) proposed RMP changes to mitigate emerging resistance risks.

The Cotton Research and Development Corporation (RDC) works with Cotton Australia to identify research needs, requests research proposals from academic and government agency researchers, and undertakes commissioning projects directly with experts. This research is partly funded by a mandatory levy imposed on growers that is matched by the Australian Government. The registrant generally does not contribute funds to the industry investments but often collaborates by running parallel programs.

A dedicated extension team supported jointly by Cotton Australia, Cotton RDC and the national cotton seed distribution company (CSD) interacts with researchers to develop and promote resistance management strategies to growers and private consultants. The registrant also works extensively with stakeholders to promote implementation of IRM.

### Brazil (Fig. 3)

The National Technical Biosafety Commission (CTNBio) assesses biosafety of genetically modified organisms for human, animal, and environmental health (CTNBio, 2018). CTNBio does not evaluate or regulate issues related to resistance management, and IRM is not mandatory. Bt crops are under the Pesticide Law, and like other pesticides follow requirements from the Ministries of Agriculture, Health and Environment for testing and registration (Fontes 2003; CTNBio 2018). For key pests, registrants in cooperation with academic research scientists have proposed IRM, established baseline susceptibilities, and are monitoring resistance (IRAC 2015).

**Fig. 3:**
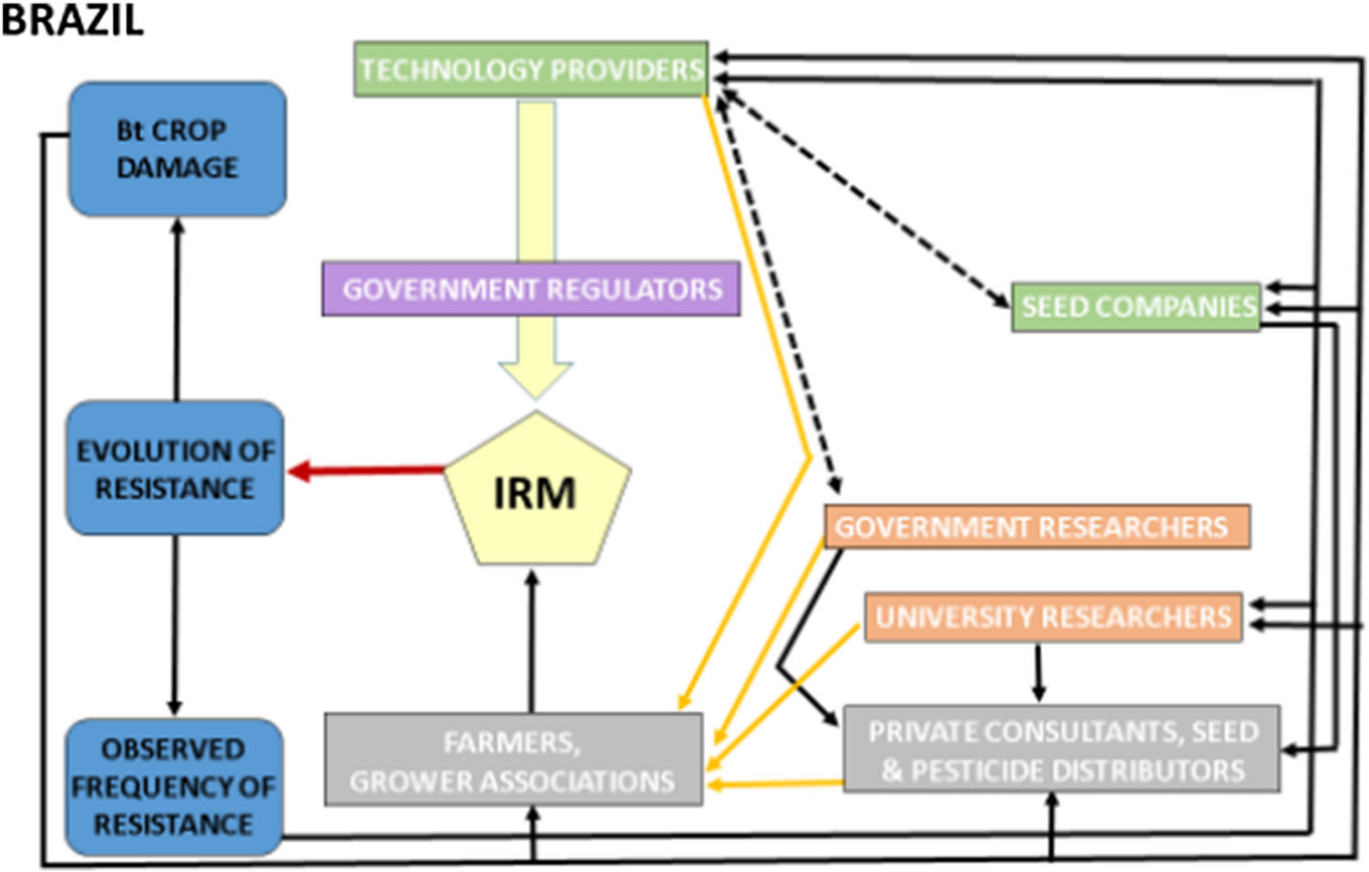
Influence diagram for Bt resistance management in Brazil. In contrast to Australia (Fig. 2), Brazil is characterized by a lack of mandates for IRM and remedial actions, and fewer linkages. See legend of Fig. 2 for details

Resistance to Bt crops in fall armyworm, *S. frugiperda*, and problems caused by the recently detected *H. armigera* have resulted in severe economic losses to Bt and other crops (Appendix S2). To address these acute problems, growers, grower associations, and private consultants in the Cerrado region formed a group to initiate IRM in consultation with industry and researchers. This initiative, formalized in 2014 by the Ministry of Agriculture as the Technical-Scientific Group for Resistance Management (GTMR), produced few tangible results. IRAC Brazil and IRAC International, which are technical groups responsible for providing a coordinated industry response to resistance problems, later outlined recommendations for management of resistance to insecticides and Bt crops in Brazil (IRAC 2015). Although these recommendations for Bt crops are actively promoted by technology providers (MAPA 2018, CIB 2018), the extent of their adoption by growers remains unclear and could be as low as 20% (Fatoretto et al. 2017). At the end of 2018, the Ministry of Agriculture recommended the use of refuges for delaying evolution of pest resistance to Bt crops across the country and requested technical information from registrants for use of such refuges (http://www.in.gov.br/materia/-/asset_publisher/Kujrw0TZC2Mb/content/id/56640378).

### India (Fig. 4)

The Genetic Engineering Appraisal Committee (GEAC) under the auspices of the Ministry of Environment, Forests and Climate Change is responsible for registration of Bt crops (http://www.envfor.nic.in/divisions/csurv/geac/bgnote.pdf). For registration of Bt cotton, registrants must provide data on (1) agronomic performance and nontarget effects of Bt crops, (2) pest biology and baseline susceptibility to Bt toxins in key target pests, and (3) IRM strategies for the key target pests (Ghosh 2001). Registrants are also required to monitor resistance evolution in *H. armigera* and implicitly in other key pests of cotton and to develop education material on Bt cotton for growers. The GEAC provides temporary registration of Bt cotton hybrids, implying that periodic evaluation of conditions for registration could lead to their withdrawal (http://www.moef.nic.in/division/genetic-engineering-approval-committee-geac).

**Fig. 4:**
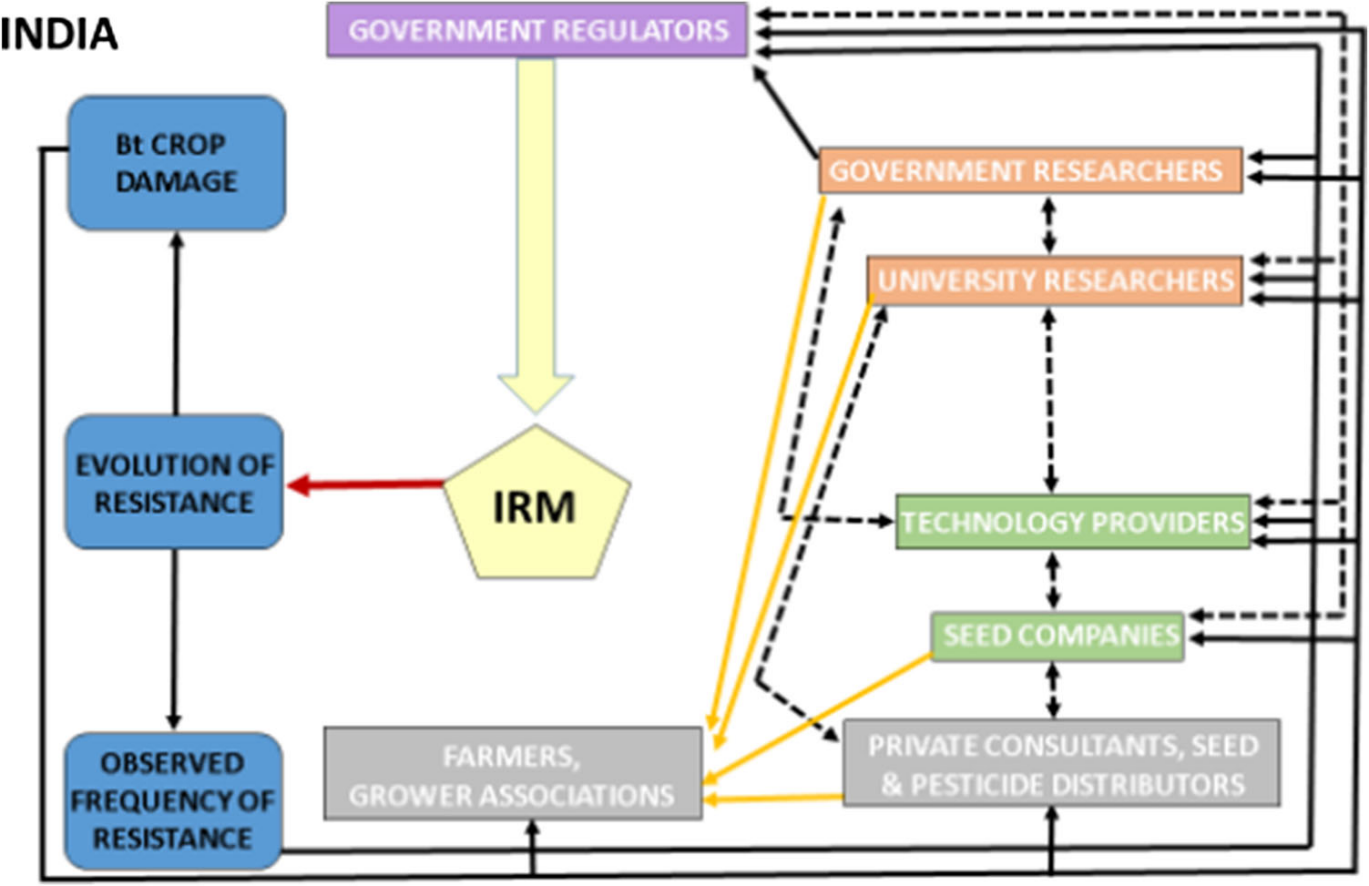
Influence diagram for Bt resistance management in India. In contrast to Australia (Fig. 2), India is characterized by a lack of auditing for IRM and mandates for remedial actions, and fewer linkages. See legend of Fig. 2 for details

The GEAC mandates use of refuges for Bt cotton (http://www.moef.gov.in/sites/default/files/geac/bgnote.pdf). To promote refuge planting, approved seed companies supply non-Bt cotton or pigeon pea seed with purchased Bt seeds, (Karihaloo and Kumar 2009), although unapproved seed companies typically do not (Section “Attributes of agricultural landscapes and crops”). Bt and refuge seed are dispensed to growers in separate bags, and registrants are not required to monitor or report data on refuge adoption to the GEAC. Most growers do not plant refuges, because this practice increases the complexity of production, growers fear a loss of profit, or unauthorized seed providers only sell Bt cotton seed (Global AgriSystem 2016; Kranthi et al. 2017).

Many stakeholders work together to promote implementation of IRM. However, implementation of IRM is challenged by many factors, including a large number of resource-poor growers planting Bt cotton (> 7.2 million), regional diversity, inadequate education, and infrastructure, and unpredictable returns. Furthermore, implementation of IRM differs across states because agriculture is under state jurisdiction, and this has caused problems with use of unauthorized Bt cotton seeds (Section “Attributes of agricultural landscapes and crops”). In response to the acute and widespread resistance crisis in pink bollworm (Appendix S3), a campaign to educate growers about IRM and IPM practices was undertaken (ISAA 2018). Since 2018, government agencies have promoted seed mixtures in which non-Bt cotton seeds (5–10%) are mixed with seeds of pyramided Bt cotton (http://seednet.gov.in/SeedGO/2016/173355_2016.pdf).

### USA (Fig. 5)

EPA mandates resistance management strategies by stipulating requirements to be met by registrants before and after registration. Product registrations are generally time-limited and conditional on registrants meeting these requirements (Matten et al. 2012). EPA does not enforce refuge mandates directly with growers. Rather, registrants or seed licensee companies use EPA-required contractual agreements with growers to enforce these mandates. Registrants are required to cancel agreements with growers found to be out of compliance with refuge requirements.

**Fig. 5:**
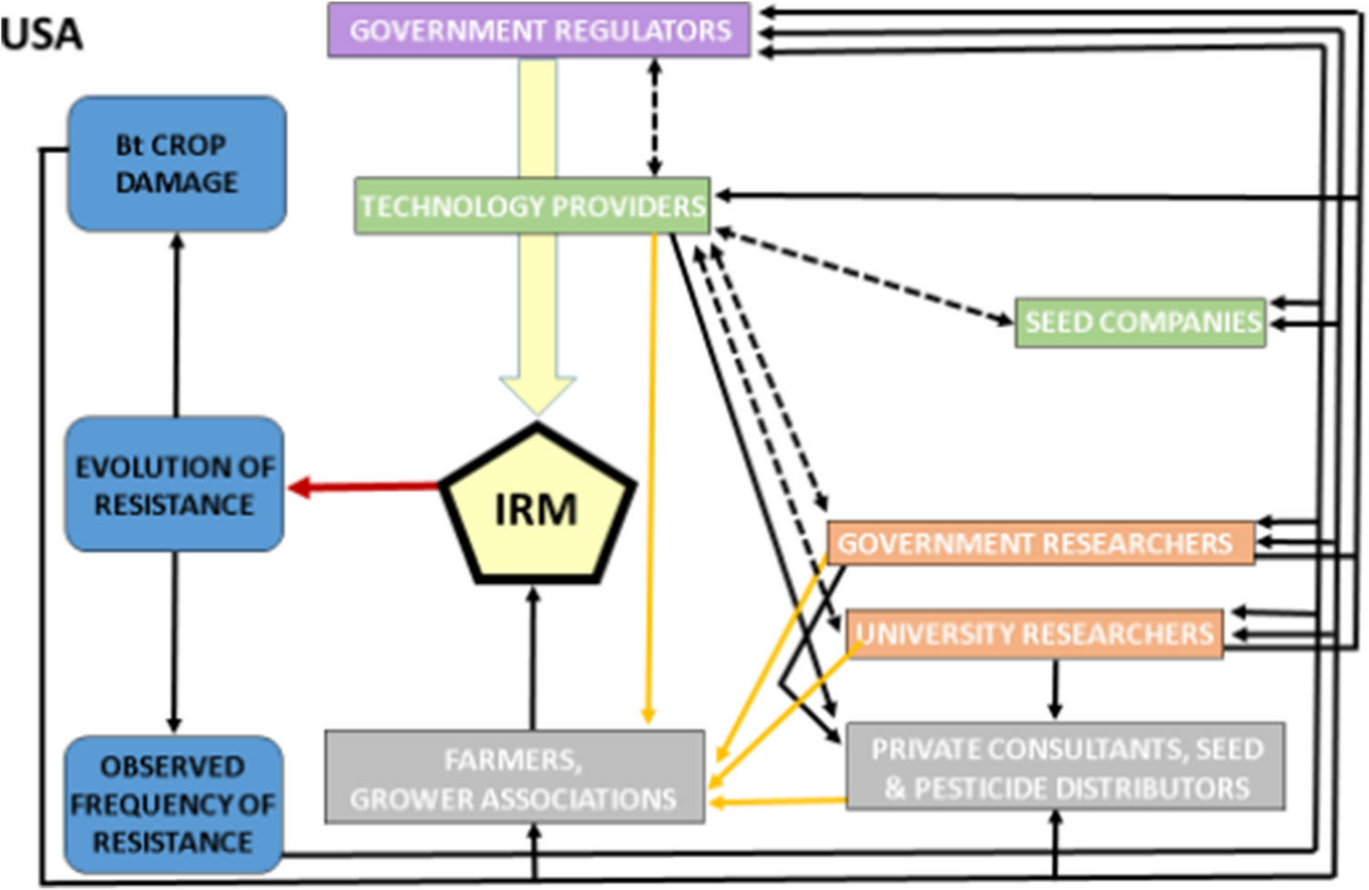
Influence diagram for Bt resistance management in the USA. In contrast to Australia (Fig. 2), the USA regime is characterized by lower participation of grower associations in enforcement of mandates for IRM and fewer linkages. See legend of Fig. 2 for details

Before registration, EPA requires information from registrants on: (1) the biology and ecology of target pests, (2) susceptibility of target pests to Bt toxin(s), (3) the mode of action of toxins, inheritance of resistance, and potential for cross-resistance, and (4) resistance management strategies for key target pests. Post-registration requirements include: (1) educating growers about resistance management, (2) monitoring grower compliance with IRM requirements, (3) monitoring changes in resistance to Bt toxins in target pests, and (4) developing and implementing remedial action plans to address cases of practical resistance. Registrants provide annual reports on post-registration conditions to EPA (Matten et al. 2012). EPA integrates information on pre- and post-registration conditions when assessing re-registration (Matten et al. 2012).

Registrants gather information requested by EPA directly or fund research conducted by scientists from academia and governmental agencies to acquire such information. Registrants work extensively with growers, grower associations, private consultants, and seed distributors to promote implementation of resistance management strategies. Academics also interact with growers, grower associations, and private consultants to develop and promote resistance management strategies.

## Opportunities for resistance management

Using the diagrams and information from previous sections, here we compare resistance management regimes among countries to outline opportunities for improving resistance management. We discuss the consequences of missing linkages among participants and the importance of matching incentives to heterogeneous social, economic, and ecological contexts.

### Fostering linkages among participants

Pest susceptibility to Bt crops was declared a public good by governments in Australia, India and the USA. Accordingly, government agencies (India and USA) or industry-led associations (Australia) mandate conditions for registering Bt crops. Brazil’s voluntary use of IRM contrasts with other cases. Standard open-access resource theory and experience with voluntary IRM for herbicide resistant crops in the USA (NRC 2010) predict suboptimal investment in stewardship of resistance management in such case.

In contrast to India, conditions for registration of Bt crops in Australia and the USA include auditing compliance with IRM requirements. In India, low grower compliance with refuge mandates has facilitated rapid evolution of resistance to Bt cotton in pink bollworm. These differences indicate that compliance monitoring is an essential component of IRM programs. However, effective audits are difficult, given the large number of cotton growers in India. In hindsight, a more robust approach for IRM in this situation could have been to release a seed mixture of pyramided Bt cotton and non-Bt cotton from the start (Section “Actors and action situations”). However, given the time it takes to test new cultivars and receive commercial approvals, waiting for availability of pyramided Bt cotton would have entailed a delay of several years in launching Bt cotton. Such delay would have decreased opportunities for reducing insecticide sprays and improving socioeconomic conditions of smallholder farm households (Kathage and Qaim 2012). Although the first Bt crops launched in almost all countries produced a single Bt toxin, this can now be avoided because pyramided Bt crops are common.

Assessment of the risk of resistance for Bt crops is inherently uncertain because of the multiple biological and socioeconomic drivers discussed above. Accordingly, effective society–biology loops are required to adjust IRM strategies and implement remedial actions when needed (Section “Institutional analysis for Bt resistance governance”). In both Australia and the USA, such loops are in place to rapidly change IRM mandates and implement remedial actions, as compelled by the Peak Industry Body in Australia and EPA in the USA. By contrast, voluntary changes in IRM and implementation of remedial actions have been slow in Brazil and India.

Prospects for effective management are enhanced by efficient coordination among participants. Such coordination, however, does not need to be fostered primarily through government actions. In Australia where implementation of IRM has been very successful (Appendix S1), an association of cotton growers advised by a broad coalition of stakeholders was granted autonomy by government authorities to oversee resistance management issues. This grower-centric approach likely facilitates implementation of IRM and remedial actions, compared to the regime in the USA where the lack of direct ties between EPA and growers results in an indirect enforcement system under the responsibility of technology providers. Nevertheless, even with significant investments in stewardship, there is no guarantee of successful outcomes, as shown in the USA where growers in many regions exhibit poor compliance that has led to resistance problems (Jaffe 2009; Reisig 2017; Tabashnik and Carrière 2017). This indicates a need to supplement some existing governance systems with new types of policies and incentives.

### Matching incentives to context

Here we explore potential leverage points and incentives for actors’ to invest in IRM. This expands on the “carrots and stick” approach advocated for weed resistance management by Barrett et al. (2016) and includes incentives reviewed by Lefebvre et al. (2015) for adoption of IPM. Carrots provide positive incentives for adoption of IRM while sticks make access to valuable resources contingent on adoption of IRM (Table 2). In principle, IRM decisions could be influenced by a wide range of monetary and nonmonetary incentives, including direct price-based instruments, such as taxes on growers’ purchase of Bt seed or subsidies on refuge seed. Non-monetary factors related to trust and social capital also influence behaviors (Ostrom 1990), underlying strategies such as social marketing and moral suasion.

**Table 2:** Positive (carrot) and negative (stick) incentives for Bt resistance management

Type of incentive	Growers	Technology providers	Researchers and Extension	Government
Carrots	Direct subsidies for IRM Discounts for refuge seed Conditional discounts to crop insurance for IRM Local goodwill from sustainable use of Bt (activated by social marketing) Tradability of refuge quota Communal refuges Good quality refuge seed (reduces cost of refuges relative to Bt crop) Better health and safety of self, coworkers, and family Reputation/pride	Rewards for biotech companies with high-performing stewardship programs (e.g., expedited permitting, extended patent life) Public–private partnership resources for resistance research and monitoring Improved public, customer relations from sustainable stewardship of technology Removing obstacles to inter-company coordination of IRM	Research funding for IRM Professional rewards, promotion for grower enrolment in extension IRM programs Support for projects that include stewardship components Recognition for roles on IRM expert panels (promotion, impact factors, respect, continued funding support)	Resources for inter-agency coordination on IRM policy Revenue from collection of Bt resistance taxes or noncompliance fees Political capital from popular IRM stewardship policies Increased resources for enforcement of IRM Outsource responsibility and capability to peak industry body
Sticks	Additional user fee or tax on Bt seed IRM conditions in eligibility for crop insurance or general agricultural subsidies Restricted future access to Bt seed (e.g., for noncompliance with refuge mandates) Reduced biofuel subsidies for corn ethanol (to incentivize crop rotation)	Rejected re-registration of Bt seed due to refuge noncompliance Fines for noncompliance with IRM regulations Public, grower outcry over unsustainable development, and deployment of technology Requirement of public disclosure of stewardship performance	Require a stewardship component in some publicly funded research projects on IRM Require publicly funded researchers to sit on IRM expert panels Require publicly funded researchers to cooperate/collaborate with scientists employed by technology providers	Threat of litigation from nonenforcement of existing laws Public outcry over poor IRM policy Threat of poor reputation

Technology providers may more easily accept the costs of stewardship for Bt crops in a monopolistic situation (Sexton et al. 2007). This may have been a factor favoring development of a successful IRM regime in Australia, although strong political will derived from past resistance crises to insecticides in *H. armigera* played a critical role (Wilson et al. 2018). In South Africa where a single technology provider was the sole registrant of Bt corn in the early years it was introduced, low refuge adoption resulted in rapid evolution of resistance in *Buseolla fusca* (Kruger et al. 2009), indicating that market power over the technology was not sufficient to delay resistance. However, in general we expect increased competition among technology providers to reduce intrinsic incentives for IRM (Sexton et al. 2007), suggesting a role for policies to incentivize stewardship in such settings (Table 2). Information-based policies can also encourage stewardship. For example, in the USA cross-company compliance data are compiled (e.g., through ABSTC) and released publicly (e.g., through the National Corn Growers Association), although compelling technology providers to publicly disclose their own stewardship performance metrics, such as overall grower refuge compliance, could help establish a more efficient stick for incentivizing IRM. Finally, finding ways to facilitate coordination of resistance management activities among technology providers without infringing on principles of free-market competition could enhance stewardship (Section “Rules”).

Policy incentives are also relevant to participants in research and extension. For research, funding is the most obvious carrot. However, a complementary stick in some contexts could be to require a stewardship component in research proposals related to pest control and crop protection. For extension, incentives could include using enrolment of growers in IRM programs as a performance metric in promotion. For research and extension, other valuable incentives could encourage service on panels overseeing stewardship for Bt crops (Table 2; Appendix S1).

Government actors might improve resistance governance by enabling inter-agency coordination. For example in the USA, using agricultural subsidies or crop insurance to incentivize growers’ IRM practices would require coordination between the USDA, whose mission is to support agriculture, and the EPA, whose mission is to enforce environmental law. Reconciling these divergent missions is important for inter-agency cooperation, but would require congressional action and new resource commitments.

Understanding the biological and institutional context of resistance management can improve policies. For instance, Ambec and Desquilbet (2012) propose that it is necessary to consider pest mobility and heterogeneity in grower exposure to pest damage to determine if a refuge mandate or a price-based incentive is more economically efficient for managing resistance. They found that with highly mobile pests, price-based incentives are more likely to constitute efficient policy, whereas lower pest mobility and less heterogeneity in exposure to pest damage favor mandates. Effectiveness of mandates over other policies is also linked to enforceability. For example, mandates are difficult to enforce among the many small growers in India due to complexity and costs.

As with other CPRs such as fisheries (Clark 2010), the regulatory costs of mandates could be reduced by allowing growers to trade refuge quotas. For example, one grower with excess refuge could sell to a grower who found it too costly to plant enough refuge to comply with the mandate (Brown 2018). However, such a trading system would need to comply with prescriptions of the specific distance of refuges from fields of Bt crops. The use of a communal refuge providing susceptible insects for Bt crop fields planted in its vicinity by several growers could also facilitate adoption of refuges in certain situations (e.g., regions with small farms such as in India).

Political economies also inform the appropriate mix of incentives for different contexts, as recognizing who gains and who loses from the carrots and sticks is critical for policy development. Ensuring gains for particular stakeholders can expedite a given policy package through the political process, although equity implications are also an issue. A hypothetical example of this might be to institute a ‘feebate’ for refuges, in which the government imposes fines on growers who planted refuge below a mandated level, but provides a subsidy to growers planting refuge above this level. If the total subsidy paid was greater than the revenue from the total fine amounts, there could be a net transfer to growers as a whole, presumably helping to reduce their political opposition to the policy.

## Conclusions

We have demonstrated the complex contextual variation around the world that precludes simplistic policy application of standard prescriptions for managing resistance to Bt crops. To address and clarify this complexity, we adapted the IAD framework to pinpoint qualitative differences among countries, which generates concrete avenues and hypotheses for future policy development. As described here, some countries developed integrated governance systems for management of resistance to Bt crops and formally mandate IRM practices, while others use voluntary actions. However, mandates for IRM appear more effective than voluntary actions. Outcomes are also critically dependent on awareness by government regulators and other stakeholders of the resistance risks associated with use of Bt crops, and the capacity to translate that awareness into efficient stewardship. We have shown that safety net measures in the form of society-to-biology loops must be built into the Bt resistance governance process to address uncertainty in resistance outcomes. Such loops encompass four activities: (1) sensitization of growers to resistance risks, (2) monitoring of grower compliance with IRM requirements, (3) monitoring of increases in resistance, and (4) implementation of remedial actions, ideally before practical resistance occurs. The comparison between Australia’s governance system and other countries also indicates that strong, direct linkages between the group that appraises resistance risks and growers appear to facilitate adoption of IRM. Nevertheless, in some cases, adoption of IRM remains inadequate even with elaborate investments in governance systems, indicating a need for further context-dependent private and public incentives for participants.

We have shown that Australia and the USA maintain sophisticated governance systems enforcing the four safety net activities mentioned above, unlike India where the large number of growers hinders monitoring of grower compliance with IRM requirements. In countries where socioeconomic conditions impede stewardship, it is therefore relevant to consider robust resistance management strategies that alleviate the need for grower compliance. Based on our review of the Indian system and recent events in China (Section “Biophysical conditions”), we propose that this could be accomplished by proactively and exclusively marketing a mixture of pyramided Bt cotton with refuge seed, which ensures compliance with refuge mandates. Universal adoption of seed mixtures would not be optimal in most countries with adequate capacity to incentivize resistance management, because the success of this strategy may be crop-specific and it may accelerate resistance evolution relative to extensive adoption of external refuges for pests with mobile larvae (Section “Actors and action situations”). With greater use of transgenic crops in lower- than higher-income countries since 2012 (James 2017), and possible introduction of Bt corn in several African countries to fight substantial corn damage by the recently introduced fall armyworm (Prasanna et al. 2018), development of robust IRM programs for Bt crops is an increasing priority. Development of new systems of cooperative management is required to enhance resistance governance (Living with Resistance Project 2018). As with medical concerns over resistant infectious disease organisms, the growing practical and ethical needs of sustaining appropriate and critical food and fiber production systems call for shared socioecological work among nations to protect susceptibility as an enduring pillar of agricultural systems.

## Electronic supplementary material

Below is the link to the electronic supplementary material.

Supplementary material 1 (PDF 637 kb)
