# Supplementary material for: Governing evolution: A socioecological comparison of resistance management for insecticidal transgenic Bt crops among four countries
Source: Ambio. 2019 Mar 21;49(1):1–16. doi: 10.1007/s13280-019-01167-0 (PMC6889122; doi:10.1007/s13280-019-01167-0)
Supplement: Supplementary file 1 — Supplementary material 1 (PDF 637 kb) [file 13280_2019_1167_MOESM1_ESM.pdf]

Ambio

Electronic Supplementary Material

Title: Governing evolution: a socio-ecological comparison of resistance management for insecticidal transgenic Bt crops among four countries

Fig. S1 Side-by-side influence diagrams for Bt resistance management in (a) Australia, (b) Brazil, (c) India and (d) the USA. Red arrow indicates a reduction in evolution of resistance following implementation of IRM. Yellow arrows indicate stakeholders that directly promote implementation of IRM by growers. Black arrows indicate the influence of a stakeholder on actions taken by other stakeholders, of a variable on another variable, or of a variable on actions taken by stakeholders (without implying whether this influence is positive or negative). Double-pointed dashed black arrows indicate mutual influence between stakeholders. The larger yellow arrow indicates significant influence of a stakeholder group on IRM. A thick border around the IRM shape indicates an audited mandatory strategy. For simplicity, we grouped “Farmers and Grower Associations” and “Private consultants, seed and pesticide distributors”, although in reality the individual entities do not necessarily always operate in the same fashion with respect to IRM governance.

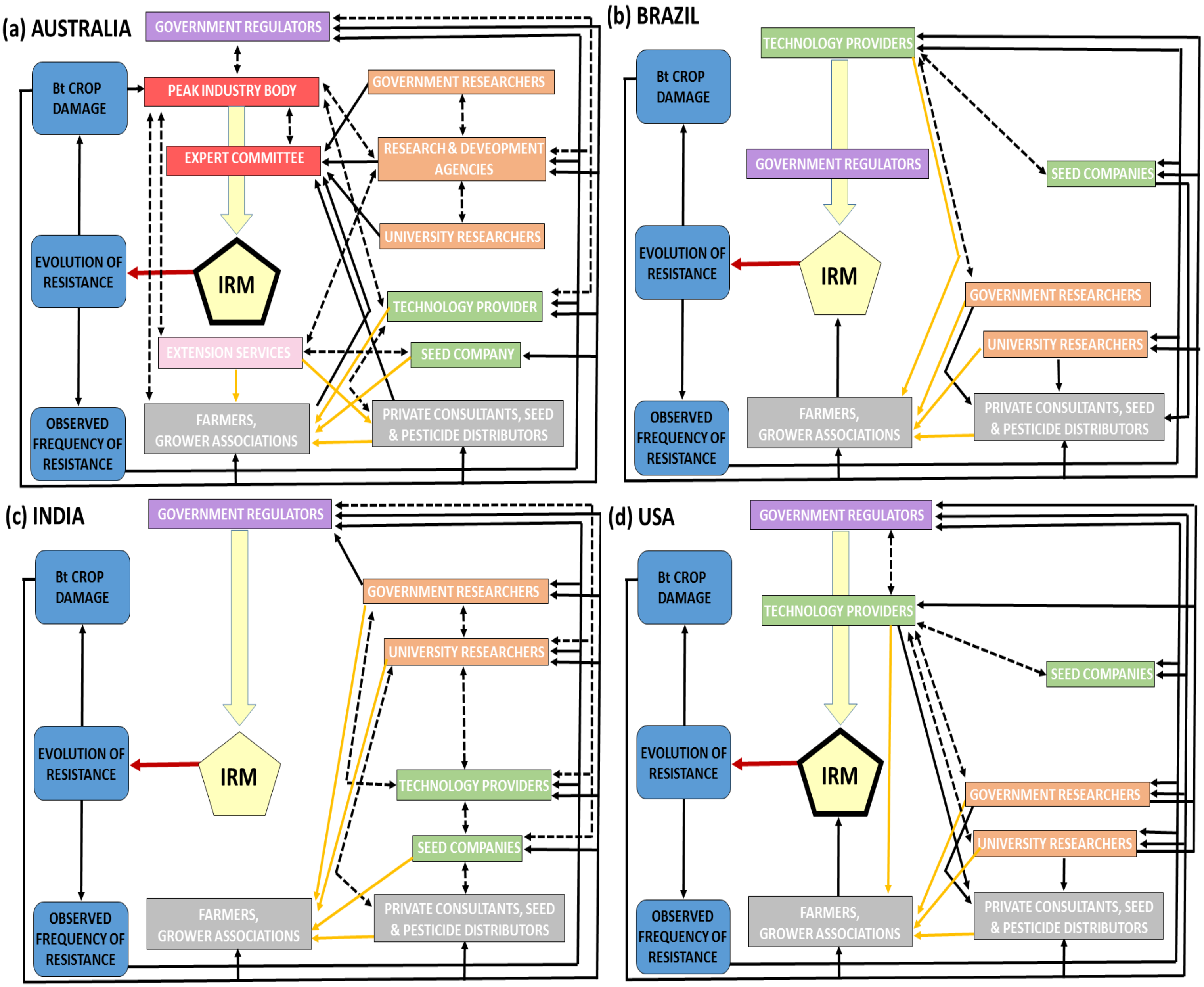
(a) AUSTRALIA
GOVERNMENT REGULATORS
Bt CROP DAMAGE
PEAK INDUSTRY BODY
GOVERNMENT RESEARCHERS
EXPERT COMMITTEE
RESEARCH & DEVEOPMENT AGENCIES
UNIVERSITY RESEARCHERS
EVOLUTION OF RESISTANCE
IRM
TECHNOLOGY PROVIDER
EXTENSION SERVICES
SEED COMPANY
OBSERVED FREQUENCY OF RESISTANCE
FARMERS, GROWER ASSOCIATIONS
PRIVATE CONSULTANTS, SEED & PESTICIDE DISTRIBUTORS
(b) BRAZIL
TECHNOLOGY PROVIDERS
Bt CROP DAMAGE
GOVERNMENT REGULATORS
SEED COMPANIES
EVOLUTION OF RESISTANCE
IRM
GOVERNMENT RESEARCHERS
UNIVERSITY RESEARCHERS
OBSERVED FREQUENCY OF RESISTANCE
FARMERS, GROWER ASSOCIATIONS
PRIVATE CONSULTANTS, SEED & PESTICIDE DISTRIBUTORS
(c) INDIA
GOVERNMENT REGULATORS
Bt CROP DAMAGE
GOVERNMENT RESEARCHERS
UNIVERSITY RESEARCHERS
EVOLUTION OF RESISTANCE
IRM
TECHNOLOGY PROVIDERS
SEED COMPANIES
OBSERVED FREQUENCY OF RESISTANCE
FARMERS, GROWER ASSOCIATIONS
PRIVATE CONSULTANTS, SEED & PESTICIDE DISTRIBUTORS
(d) USA
GOVERNMENT REGULATORS
Bt CROP DAMAGE
TECHNOLOGY PROVIDERS
SEED COMPANIES
EVOLUTION OF RESISTANCE
IRM
GOVERNMENT RESEARCHERS
UNIVERSITY RESEARCHERS
OBSERVED FREQUENCY OF RESISTANCE
FARMERS, GROWER ASSOCIATIONS
PRIVATE CONSULTANTS, SEED & PESTICIDE DISTRIBUTORS

S1 Context of resistance management for Bt crops in Australia

In Australia single-toxin Bt cotton (Cry1Ac) was introduced in 1996 and withdrawn from the market in 2005, two years after a complete transition by industry to dual toxin Bt cotton (Cry1Ac + Cry2Ab). Three-toxin Bt cotton (Cry1Ac + Cry2Ab + Vip3A) was introduced in 2017 and completely replaced dual toxin Bt cotton in 2018. Neither of the two primary target pests (*H. armigera* and native budworm/Australian bollworm, *H. punctigera*) has evolved practical resistance to Bt crops (Downes and Mahon 2012a,b; Tabashnik and Carrière 2017). However, a rapid increase in resistance frequency to Cry2Ab in *H. punctigera* in 2008/09 (Downes et al. 2010) stimulated the TIMS committee to develop a contingency plan for mitigating resistance to the toxins within dual toxin Bt cotton (Downes et al. 2010).

Bt crops themselves are regulated by the Office of the Gene Technology Regulator (OGTR), but the toxins they produce are regulated by the Australian Pesticides and Veterinary Medicines Authority (APVMA). As part of its responsibility for upholding the *Gene Technology Act 2000,* the OGTR evaluates and manages the risks posed by a Bt crop with a view to protecting the health and safety of people and the environment and subsequently issues a plan to mitigate risks associated with the gene technology, which may include licensing conditions for registrants (Agricultural Biotechnology Council of Australia, 2017). After Cry1Ac cotton was registered in 1996, the APVMA worked with the Peak Representative Body representing the Australian cotton growers (known currently as Cotton Australia, hereafter CA) and Monsanto to ensure its appropriate resistance management. CA is led by a Board of nine cotton growers and ginners. Established in 1972 (as the Australian Cotton Foundation), CA merged with the Australian Cotton Growers Research Association (ACGRA) in 2008 to provide a united voice

for cotton growers across research, stewardship, natural resource management and cotton production issues.

Before introducing the first commercial *Bt* transgenic cotton in 1995, the ACGRA formed a broadly representative, grower led Advisory Committee called the Transgenic and Insect Management Strategies committee (TIMS). This industry expert committee was established to oversee the development of resistance management strategies for insecticides and *Bt* cotton and later herbicide resistant cotton traits. TIMS is now facilitated by CA and the Cotton Research and Development Corporation (CRDC, a statutory authority established in 1990 by the Australian Government; see below) and functions as a cotton industry stewardship group, with broad representation from growers, research organizations and members of the pulse and grains industries. It also includes consultants that typically subscribe to Crop Consultants Australia (CCA), an association that provides a forum for information exchange, networking, professional development and technical training of agronomists, and contribute a background in providing advice to Australia’s cotton, grain, pulse, and oilseed producers. TIMS speaks for the whole cotton growing industry by assigning representatives from a regional distribution to specific areas of responsibility. It also provides a forum for management issues to be discussed across relevant industries to consider joint strategies.

Since its representatives are independent from commercial providers and must declare potential conflicts, the TIMS committee is considered an independent body capable of reviewing and researching market sensitive data. It develops and maintains a close relationship with the APVMA, OGTR and state government control-of-use agencies which request advice, and require endorsement, from them pertaining to applications from registrants for permits, resistance management plans and their amendments. Current and future registrants approach TIMS with

‘market sensitive’ issues pertaining to resistance management, which allows the group to maintain a holistic view.

While the Resistance Management Plan (RMP) for Bt cotton is supported by industry, it is also a regulatory requirement of the registrant. It is based on 3 key strategies: (1) planting during a restricted period to minimize exposure, (2) growing refuges, and (3) removing resistant individuals at the end of the cotton season. Farmers that plant Bt cotton seed sign a Technology Users Agreement (TUA) with the registrant which requires them to adhere to the RMP. The registrant is responsible for auditing planting dates (all farms), refuges (location, area, mid-season quality – all farms; early season quality – ~6% of TUA’s randomly selected) and end of season destruction of pest populations (timing, quality – all farms) and reports farmer compliance annually to the TIMS Bt Technical Panel and the APVMA. Breeches of the RMP that are considered beyond the reasonable control of growers are alleviated with a Remedial RMP targeted at compensating a deficiency in one tactic with a heightened application of another. The registrant reserves the right to prevent future use of their product by growers that appear to have deliberately breeched the RMP.

Annually, between growing seasons, the TIMS committee contributes to the development and/or amendment of the RMP for Bt cotton. It is advised of the scientific merits of all proposed strategies by a technical panel, comprised primarily of appointed cotton industry research experts in resistance management and IPM. The recommendations from the TIMS Bt Technical Panel are based on reviews of proposed and existing strategies from technology providers in light of their resistance plan compliance reports, as well as any new research findings or recent information on resistance frequencies. The TIMS committee considers the trade-off between

practicality and socioeconomic impact versus the science behind the most effective response to current resistance risks (Kauter et al. 2009).

The CRDC works with CA to identify priority questions relating to managing resistance to Bt cotton and currently calls annually for tenders from academic and government agency researchers and commissions projects directly with specific experts. This research is partly funded by growers, which are required to contribute an annual levy towards the investment that is matched by the Australian Government. The registrant does not contribute funds to the CRDC investments, but often collaborates with researchers by running parallel programs that extend the scope of their projects. For instance, since the introduction of Bt cotton, the CRDC invested in a program to monitor resistance to its toxins and the registrant runs a parallel program that is reported at industry forums alongside the results from independent researchers (Mahon et al. 2007; Downes and Mahon 2012a,b; Downes et al. 2016). The registrant could also conduct research on Bt cotton outside of the scope of projects tendered by the CRDC (e.g., Knight et al. 2013, 2016), and may consult with key researchers within the Bt Technical Panel to develop projects. From 2005 to 2012, Cooperative Research Centres (CRC) grant programs supported collaborative partnerships between industry and researchers that emphasized adapting the cotton IPM system to a changed pest complex resulting from the introduction of Bt cotton (Wilson et al. 2013).

Before 2012 key messages from resistance management research were delivered to industry by state government research agencies and subsequently a dedicated effort from the CRC with input from CRDC. In 2012 CottonInfo became the cotton industry’s extension program supported jointly by CA, CRDC and the national cotton seed distribution company, CSD (Cotton Seed Distributors Ltd, a not-for-profit formed in 1967 by farmers). The team consists of regional

extension officers for on-the-ground support, as well as technical experts in Bt cotton stewardship and IPM which are the point of contact to the wider research community and provide in-depth analysis and research to farmers, consultants, and agronomists. Strategic extension activities that involve exchange of information between key stakeholders in the cotton industry and resistance-based research include articles in tailored magazines, newsletters, industry publications, reports on resistance frequencies during and post-season, research presentations as part of industry-wide roadshows, CCA seminars, resistance forums, farmer conferences, field days, and formal workshops and education courses.

In particular, the CRDC and the TIMS committee facilitated since 2006 an annual review of Bt resistance research and extension activities as part of a forum meeting. Led by the TIMS Chair, CA and CRDC representatives, the forum brings together researchers, growers, consultants, and representatives from the registrant and the industry CottonInfo team, to discuss research project progress and communication on *Bt* resistance. This forum is a key precursor of the annual review of the RMP for Bt cotton by the TIMS committee.

The CottonInfo team includes experts in the cotton industry's best management practice program, *my*BMP, an earlier iteration of which was initiated by growers in 1999 in response to public perception about environmental performance. The program sets the performance criteria and provides a framework for participation in, and accreditation of, growers. It includes a module on IPM which covers managing resistance in Bt cotton according to the RMP and more detailed terms and conditions of the TUA, as well as correct sampling and identification of pests and beneficials to make sound decisions for pest control. The *my*BMP team sits within CottonInfo, and CottonInfo encourages growers to participate in *my*BMP.

S2 Context of resistance management for Bt crops in Brazil

The first Bt crop commercialized in Brazil was Cry1Ac cotton in 2005. Following this, CTNBio approved practically all Bt technologies submitted for registration (Table S1) (CTNBio, 2018).

Table S1. Bt crops approved for commercial release in Brazil.

Crop	Bt toxins (s)	Year
Cotton	Cry1Ac	2005
	Cry1Ac + Cry1F	2009
	Cry1Ac + Cry2Ab2	2009
	Cry1Ab + Cry2Ae	2011
	Cry1Ac + Cry2Ab2 + Vip3A	2016
	Cry1Ac + Cry2Ae +Vip3A	2017
	Cry1Ac + Cry1F + Vip3A	2018
Corn	Cry1Ab	2007
	Cry1F	2008
	Vip3Aa20	2009
	Cry1A.105 + Cry2Ab2	2009
	Cry3Bb1	2010
	Cry1Ab + Vip3Aa20	2010
	Cry1A.105 + Cry1F + Cry2Ab2	2010
	Cry1Ab + Cry1F	2011
	Cry1A.105 + Cry2Ab2 + Cry3Bb1	2011
	Cry1F + Cry34/35Ab1	2013
	mCry3A	2014
	Cry1Ab + Vip3Aa20 + mCry3A	2014
	Cry1F + Vip3Aa20	2015
	Cry1Ab + Vip3Aa20	2015
	Cry1Ab + Cry1F + Vip3Aa20	2015
	eCry3.1Ab	2015
	Cry1Ab + Cry1F + Cry3A + eCry3.1Ab + Vip3Aa20	2015
	Cry1A.105 + Cry2Ab2 + Cry3Bb1 + Cry34/35Ab1	2016
	Cry1A.105 + Cry2ab2 + Vip3Aa20	2017
	Cry1Ab + Cry1A.105 + Cry2Ab2 + Vip3Aa20	2017

	Cry1A.105 + Cry2Ab2 + Cry1F + Vip3Aa20	2017
Soybean	Cry1Ac	2010
	Cry1Ac + Cry1F	2016
	Cry1A1.105 + Cry2Ab2	2017
	Cry1A.105 + Cry2ab2 + Cry1Ac	2018
Sugarcane	Cry1Ab	2017

Brazil's climate is varied and dominated by tropical conditions, implying that annual cropping systems are diverse and complex and pest pressure is high. Successive plantings of Bt maize, Bt cotton, and Bt soybean in the Cerrado region each year present a formidable challenge for managing resistance in polyphagous pests completing many generations during the long growing season and exploiting multiple Bt crops. Such pests include the fall armyworm, the soybean looper (*Chrysodeixis includens*) and the cotton bollworm (*H. armigera*). Field-evolved practical resistance to Cry1F corn was reported in fall armyworm only 4 years after release of these Bt crops (Farias et al. 2014). Because of cross-resistance, evolution of fall armyworm resistance to Cry1F has negatively affected the performance of Bt corn and Bt cotton producing other Cry1 toxins (Bernardi et al. 2015; Horikoshi et al. 2016; Omoto et al. 2016).

While Brazilian Bt corn growers have benefitted from higher yield and ease of management, most have also been negatively affected by the rapid evolution of resistance to Bt in fall armyworm (Farias et al. 2014; Omoto et al. 2016). Compounding this problem is the recent detection of the invasive species *H. armigera,* which has caused severe yield losses in some major crops (Czepak et al. 2013, Specht et al. 2013). These new problems with fall armyworm and *H. armigera* resulted in a severe economic crisis in an agribusiness sector that represented ≈ 21.5% of the Gross Domestic Product (GDP) of Brazil in 2015 (CEPEA 2016).

Brazil stands out for its lack of government mandates for resistance management. Greater involvement of participants from the public and private sector could help remediate this situation. A good example of such involvement is the case of the soybean rust, *Phakopsora pachyrhizi* (Godoy et al. 2016). Following initiatives led by a consortium of public scientists, the soybean grower association, and the private sector, adoption of soybean-free periods occurred in several states of Brazil to reduce proliferation of the pathogen and early colonization of soybean crops. The program developed by this consortium eventually metamorphosed into the National Program for Asian Soybean Rust Control launched by the Brazilian Ministry of Agriculture, Livestock and Food Supply, resulting in changes of the soybean agricultural system across Brazil (Godoy et al. 2016).

S3 Context of resistance management for Bt cotton in India

Bt cotton producing Cry1Ac and Cry1Ac + Cry2Ab was first commercialized in 2002 and 2006, respectively. Use of this pyramided Bt cotton increased rapidly and by 2012 reached ca. 90% of cotton acreage (James 2016). In India, Bt cotton targets key lepidopteran pests including the cotton bollworm, *Helicoverpa armigera* and the pink bollworm, *Pectinophora gossypiella*.

Since 1985, the Government of India has used IPM principles to guide its agricultural policy for reducing dependence on synthetic pesticides and promoting more environmentally-friendly agriculture (http://pib.nic.in/newsite/PrintRelease.aspx?relid=110364). Accordingly, a minimum refuge of 20% non-Bt cotton or five rows of non-Bt cotton, whichever is greater, is mandated to be planted around each Bt cotton field by the GEAC (http://www.moef.gov.in/sites/default/files/geac/bgnote.pdf). Despite predominance of two-toxin Bt cotton, refuge policy did not change since 2002, except for an option of planting pigeon

pea instead of non-Bt cotton as refuge. The percentage of Bt cotton planted in India has exceeded 80% since 2008 (James 2016), demonstrating that the 20% refuge strategy mandated by the GEAC has generally not been implemented.

The Directorate of Plant Protection, Quarantine and Storage, within the Ministry of Agriculture and Farmers' Welfare, does not regulate the concentration of Bt toxin(s) or efficacy of Bt cotton. However, the minimum expression of Bt toxin in leaves or seed, and the purity of Bt cotton seed is regulated under the Seed Act of 1966.

Many studies demonstrate widespread practical resistance to both Cry1Ac and Cry1Ac + Cry2Ab cotton in pink bollworm in India (Dhurua and Gujar 2011; Fabrick et al. 2014; Kranthi 2015; Desai et al. 2016; Malthankar and Gujar 2016; Mohan et al. 2016; Tabashnik and Carrière 2017), and no new Bt cotton technology will be available for several years to address this problem (Tabashnik and Carrière 2017). High pink bollworm damage to Bt and non-Bt cotton in several regions of India in 2017 led State Governments to implement novel resistance management strategies in 2018. These strategies include the planting of Bt cotton during a restricted period, promoting refuges and seed mixtures of Bt and non-Bt cotton, monitoring seed and pesticide quality, surveying pest incidence, educating farmers and interacting with different stakeholders to address the resistance crisis in pink bollworm.

Besides practical resistance in pink bollworm, some studies indicate a reduction in cotton bollworm susceptibility to Cry1Ac and Cry1Ac + Cry2Ab cotton (Gujar et al. 2007; http://www.moef.nic.in/divisions/csurv/geac/GEAC-Cry1Ac_Monitoring_report-2009-10.pdf, Ranjith et al. 2010; Hallad et al. 2014), although implications of such resistance for pest management remain to be determined. Some have proposed that natural non-cotton refuges are abundant enough to delay resistance to Bt cotton in cotton bollworm

(http://www.agbioworld.org/biotech-info/articles/biotech-art/safety-bt-cotton.html, Ravi et al. 2005). However, Singla et al. (2012) reported that such refuges were too rare in some regions to significantly reduce the risk of resistance even for two-toxin Bt cotton.

S4 Context of resistance management for Bt crops in the USA

Bt corn and cotton are commercially available the United States, where they are registered by three companies resulting from recent mergers: Bayer-Monsanto, Dupont-Dow, and ChemChina-Syngenta. The list of single and multi-toxin Bt corn targeting lepidopteran and coleopteran pests in the US is reported in Carrière et al. (2016). Since 2011, only pyramided Bt cotton has been available for the control of lepidopteran pests. Current pyramided cotton cultivars produce the toxins Cry1Ac + Cry1F, Cry1Ac + Cry2Ab, Cry1Ab + Cry2Ae, Cry1Ac + Cry1F + Vip3Aa, and Cry1Ac + Cry2Ab + Vip3a.

Since the introduction of Cry1Ab corn and Cry1Ac cotton in 1996, four pests have evolved practical resistance to Bt crops in the USA (Tabashnik and Carrière 2017). *Helicoverpa zea* evolved practical resistance to Cry1Ac cotton in some southeastern states six years after introduction of this crop (Tabashnik et al. 2008, 2009). The economic impacts of this resistance was initially mitigated by introducing pyramided Bt cotton producing Cry1Ac + Cry2Ab and use of insecticides, although rapid evolution of *H. zea* resistance to Cry2Ab followed the release of this pyramid (Tabashnik et al. 2009, 2013). *H. zea* also evolved practical resistance to Cry1Ab and Cry1A.105 + Cry2Ab corn in Maryland, which resulted in selection of other Bt hybrids by farmers or greater use of insecticides (Dively et al. 2016). The fall armyworm evolved practical resistance to Cry1F corn in only three years in Puerto Rico, which led to voluntary withdrawal of this Bt crop by industry (Storer et al. 2012; Tabashnik et al. 2013). Western corn rootworm also

rapidly evolved practical resistance to Cry3Bb, mCry3Aa, Cry3.1Ab, and Cry34/35Ab produced in Bt corn in some states of the Corn Belt (Tabashnik and Carrière 2017). Corn rootworm was resistant to eCry3.1Ab corn before it was commercialized due to strong cross-resistance between Cry3Bb and eCry3.1Ab (Carrière et al. 2015, 2016; Tabashnik and Carrière 2017). EPA recently required that registrants of rootworm-active Bt corn promote IPM measures for corn rootworm and implement resistance mitigation measures in affected fields (e.g., crop rotation) to address this situation (US EPA 2018). *Striacosta albicosta* evolved practical resistance to Cry1F corn in 10 years (Tabashnik and Carrière 2017).

The maintenance of susceptibility of target pests to Bt toxins was declared to be in the public good following the claim from the Environmental Protection Agency (EPA) that Bt crops can reduce human and environmental exposure to insecticides that are less benign than Bt (US EPA 1998, 2001; Glaser and Matten 2003; Matten et al. 2012). Sustaining pest susceptibility was also valued because rapid evolution of resistance was perceived as a threat to the widespread use of Bt sprays in organic agriculture (US EPA 1998, 2001; Walker et al. 2003; Berwald et al. 2006; Welch et al. 2015).

Bt toxins produced by transgenic crops are regulated under the Federal Insecticide and Rodenticide Act (FIFRA) as plant incorporated protectants (PIPs). During the registration process of PIPs (plant incorporated protectants), EPA evaluates whether Bt toxins can result in unreasonable harm to humans and the environment. In defining unreasonable, EPA considers social, environmental and economic costs and benefits resulting from regulatory decisions (Berwald et al. 2006). EPA includes an examination of the risk and potential consequences of evolution of resistance to the Bt toxins in target pests, non-target effects and risks of unwanted gene flow between Bt crops and related non-cultivated plants (US EPA 1998, 2001, NRC 2010,

NASEM 2016). EPA social and economic considerations include impacts on profits at the farm and industry level and farmer's ability to manage risk and quality of the crop (Berwald et al. 2006).

S5 Temporal dimension in Bt resistance management

The conventional approach to evaluating risk (e.g., in insurance) is to include risk aversion as a parameter in the decision problem or through more comprehensive approaches such as *expected utility theory*. An extreme version of risk version, sometimes called the *max-min*, is to focus on maximizing the payoff in the worst possible state of the world.

For intertemporal decision-making, the standard approach in economics of evaluating a stream of values over time is the *net present value* (NPV) criterion, in which values over time are aggregated by discounting future values at a fixed rate relative to the current value. This discount rate is often viewed by economists as the rate of return we would expect on alternative investments such as the stock market; the US Office of Management and Budget (OMB) sets this rate at 7% for valuing intertemporal benefits and costs of government projects (OMB 1992). Alternative intertemporal approaches include the *max-min* criterion akin the risk aversion case – that is, choosing the regimes with the highest *minimum* value over the relevant time horizon (Heal 2000). In an intertemporal context, this criterion is often justified on the basis of intergenerational equity: maximizing the value to the least well-off generation (Hartwick 1978). Alternatively, one could evaluate outcomes based on the value yielded in the long-run. When a biophysical outcome is used, this criterion is referred to as *maximum sustainable yield* (MSY). When an economic outcome is used, this criterion becomes *maximum sustainable economic yield* (MSEY) (Clark 2010).

To ground the discussion of the temporal dimension, Figure S2a depicts a hypothetical example with farm profits over time across four alternative policy regimes: a regime with largescale, maximal Bt deployment with no refuge, Bt deployment with some refuge, Bt deployment with lots of refuge, and then finally a regime with conventional insecticide-based control instead of Bt. Panel S2b shows how each of these regimes may be more or less preferred under different evaluative criteria: NPV criterion using a range of discount rates (with a higher discount rate placing less weight on future value), the max-min criterion, and the MSEY. Criteria strongly oriented towards the present (e.g., the NPV criterion with a 7% discount rate) favor the regime with lots of Bt and no refuge. The max-min criterion favors the chemical control regime in this scenario because all the other regimes' minimum values (occurring in Year 0) are lower. The MSEY criterion favors the high refuge regime, precisely because this regime yields the highest payoff in the last period. Note the choice of time horizon also affects the preference ranking for regimes (and is in fact mathematically related to the discount rate). As the time horizon is extended towards infinity, for example, the ranking of regimes under the NPV criterion with a 0% discount rate approaches the same ranking as that implied by MSEY (Clark 2010).

Figure S2. Evaluating the temporal dimension in Bt resistance management.

a. Hypothetical farm profits over time, by regime

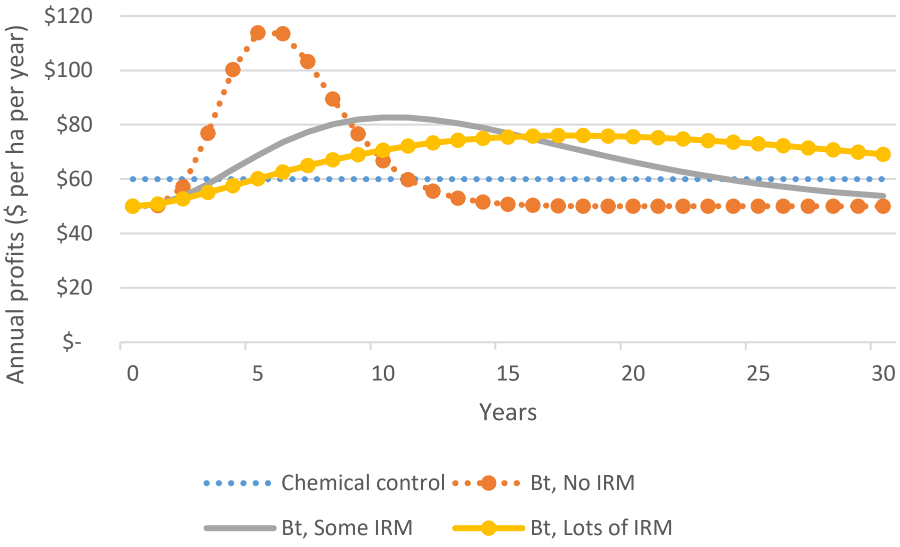

b. Hypothetical application of evaluative criteria to each regime.

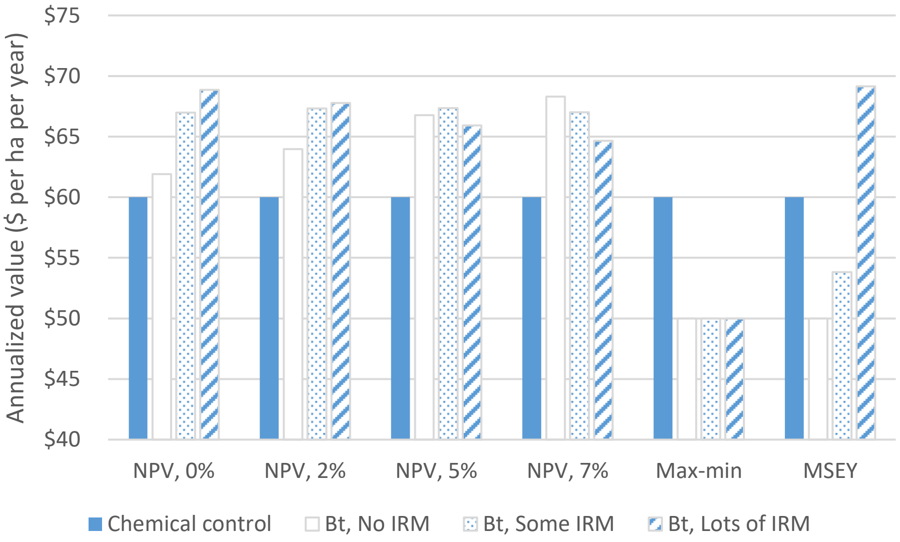

References

Agricultural Biotechnology Council of Australia. 2017. The Official Australian Reference Guide to Agricultural Biotechnology and GM Crops. Third Edition.

Bernardi, D., E. Salmeron, R.J. Horikoshi, O. Bernardi, P.M. Dourado, R.A. Carvalho, S. Martinelli, G.P. Head, et al. 2015. Cross-resistance between Cry1 proteins in fall armyworm (*Spodoptera frugiperda*) may affect the durability of current pyramided Bt maize hybrids in Brazil. *PLoS One* 10: e0140130.

Berwald, D., S. Matten, and D. Widawsky. 2006. Economic analysis and regulating pesticide biotechnology at the US environmental protection agency. In *Regulating Agricultural Biotechnology: Economics and Policy*, ed. R.E. Just, J.M. Alston, and D. Zilberman, 21-35. New York: Springer.

Carrière, Y., N. Crickmore, and B.E. Tabashnik. 2015. Optimizing pyramided transgenic Bt crops for sustainable pest management. *Nature Biotechnology* 33: 161-168.

Carrière, Y., J.A. Fabrick, and B.E. Tabashnik. 2016. Can pyramids and seed mixtures delay resistance to Bt crops? *Trends in Biotechnology* 34: 291-302.

CEPEA. 2016. Brazilian Agribusiness GDP. https://www.cepea.esalq.usp.br/en/brazilian-agribusiness-gdp.aspx

Clark, C.W. 2010. *Mathematical bioeconomics: the mathematics of conservation* (Vol. 91). John Wiley & Sons.

CTNBio 2018. Summary of GM crops deregulated in Brazil (Ministério da Ciência, Tecnologia, Inovações e Comunicações), Brasília, Edn. 08/31/2018

Czepak, C., K.C. Albernaz, L.M. Vivan, H.O. Guimarães, and T. Carvalhais. 2013. First reported occurrence of *Helicoverpa armigera* (Hübner)(Lepidoptera: Noctuidae) in Brazil. *Pesquisa Agropecuária Tropical* 43: 110-113.

Desai, D., Jitendra, K. Bahuguna, and M. Kulkarni. 2016. Seeds of decline. http://www.downtoearth.org.in/coverage/seeds-of-decline-56200

Dhurua, S., and G.T. Gujar. 2011. Field evolved resistance to Bt protein Cry1Ac in the pink bollworm, *Pectinophora gossypiella* (Saunders) (Lepidoptera: Gelechiidae), from India. *Pest Management Science* 67: 898-903.

Dively, G.P., P.D. Venugopal, and C. Finkenbinder. 2016. Field-evolved resistance in corn earworm to Cry proteins expressed by transgenic sweet corn. *PLoS ONE* 11: e0169115.

Downes, S.J., and R. Mahon. 2012a. Successes and challenges of managing resistance in *Helicoverpa armigera* to Bt cotton in Australia. *GM Crops and Food* 3: 228-234.

Downes, S.J., and R. Mahon. 2012b. Evolution, ecology and management of resistance in *Helicoverpa* spp. to Bt cotton in Australia. *Journal of Invertebrate Pathology* 110: 281-286.

Downes, S.J., R.J. Mahon, L. Rossiter, G. Kauter, G. Fitt, and G. Baker. 2010. Adaptive management of pest resistance by *Helicoverpa* species (Noctuidae) in Australia to the Cry2Ab Bt protein in Bollgard II cotton. *Evolutionary Applications* 3: 574-584.

Downes, S.J., T. Walsh, and W.T. Tay. 2016. Bt resistance in Australian insect pests. *Current Opinions in Insect Science* 15: 78-83.

Fabrick, J.A., J. Ponnuraj, A. Singh, R.K. Tanwar, G.C. Unnithan, A.J. Yelich, X. Li, Y. Carrière, et al. 2014. Alternative splicing and highly variable cadherin transcripts associated with field-evolved resistance of pink bollworm to Bt cotton in India. *PLoS One* 9: e97900.

Farias, J.R., D.A. Andow, R.J. Horikoshi, R.J. Sorgatto, P. Fresia, A.C. dos Santos, and C. Omoto. 2014. Field-evolved resistance to Cry1F maize by *Spodoptera frugiperda* (Lepidoptera: Noctuidae) in Brazil. *Crop Protection* 64: 150-158.

Glaser, J.A., and S.R. Matten. 2003. Sustainability of insect resistance management strategies for transgenic Bt corn. *Biotechnology Advances* 22: 45–69.

Godoy, C.V., C.D.S. Seixas, R.M. Soares, F.C. Marcelos-Guimarães, M.C. Meyer, and L.M. Costamilan. 2016. Asian soybean rust in Brazil: past, present, and future. *Pesquisa Agropecuária Brasileira* 51: 407-421.

Gujar, G.T., V. Kalia, A. Kumari, B.P. Singh, A. Mittal, R. Nair and M. Mohan. 2007. *Helicoverpa armigera* baseline susceptibility to *Bacillus thuringiensis* (*Bt*) Cry proteins and resistance management for *Bt* cotton in India. *Journal of Invertebrate Pathology* 95: 214–219.

Hallad, A.V., S.S. Udikeri, S.B. Patil, and A.R.S. Bhat. 2014. Field performance of first and second generation Bt cotton events for bollworm resistance in rainfed ecosystem. *International Journal of Current Microbiology and Applied Sciences* 3: 223-238.

Hartwick, J.M. 1978. Substitution among exhaustible resources and intergenerational equity. *The Review of Economic Studies* 45: 347-354.

Heal, G. 2000. *Valuing the Future: Economic Theory and Sustainability*. New York: Columbia University Press.

Horikoshi, R.J., D. Bernardi, O. Bernardi, J.B. Malaquias, D.M. Okuma, L.L. Miraldo, S.D.A. Fernando, and C. Omoto. 2016. Effective dominance of resistance of *Spodoptera frugiperda* to Bt maize and cotton varieties: implications for resistance management. *Scientific Reports* 6: 34864.

James, C., 2016. Executive Summary: Global Status of Commercialized Biotech/GM Crops: 2014, Brief 52. New York, Ithaca: *International Service for the Acquisition of Agri-Biotech Applications*.

Kauter, G., S. J. Downes, and B. Pyke. 2009. A snapshot of the Cotton Australia TIMS committee in 2009. *The Australian Cotton grower* 30: 8-11.

Knight K., G. Head, and J. Rogers. 2013. Season-long expression of Cry1Ac and Cry2Ab proteins in Bollgard II cotton in Australia. *Crop Protection* 44: 50-58.

Knight K., G. Head, and J. Rogers. 2016. Relationships between Cry1Ac and Cry2Ab protein expression in field-grown Bollgard II® cotton and efficacy against *Helicoverpa armigera* and *Helicoverpa punctigera* (Lepidoptera: Noctuidae). *Crop Protection* 79: 150-158.

Kranthi, K.R. 2015. Pink bollworm strikes Bt-cotton. In *Cotton Statistics and News No. 35*, ed. A. Singh, 1-6. Mumbai: Cotton Association of India.

Mahon, R.J., K.M. Olsen, K.A. Garsia, and S.R. Young. 2007. Resistance to *Bacillus thuringiensis* toxin Cry2Ab in a strain of *Helicoverpa armigera* (Lepidoptera: Noctuidae) in Australia. *Journal of Economic Entomology* 100: 894-902.

Malthankar, P.A., and G.T. Gujar. 2016. Toxicity of *Bacillus thuringiensis* Cry2Ab and the inheritance of Cry2Ab resistance in the Pink bollworm, *Pectinophora gossypiella* (Saunders) (Lepidoptera: Gelechiidae). *Indian Journal of Experimental Biology* 54: 586-596.

Matten, S.R., R.J. Frederick, and A.H. Reynolds. 2012. United States Environmental Protection Agency insect resistance management programs for plant-incorporated protectants and use of simulation modeling. In *Regulations of agricultural biotechnology: the United States and Canada,* ed. C.A. Worzniak and A. McHughen, 175-267. New York: Springer.

Mohan, K.S., K.C. Ravi, P.J. Suresh, D. Sumerford, and G.P. Head. 2016. Field resistance to the *Bacillus thuringiensis* protein Cry1Ac expressed in Bollgard® hybrid cotton in pink bollworm, *Pectinophora gossypiella* (Saunders), populations in India. *Pest Management Science* 72: 738-746.

National Research Council. 2010. *The impact of genetically engineered crops on farm sustainability in the United States.* Washington, DC: The National Academies Press.

National Academies of Sciences, Engineering, and Medicine. 2016. *Genetically Engineered Crops: Experiences and Prospects.* Washington, DC: The National Academies Press.

Omoto, C., O. Bernardi, E. Salmeron, R.J. Sorgatto, P.M. Dourado, A. Crivellari, R.A. Carvalho, A. Willse, et al. 2016. Field-evolved resistance to Cry1Ab maize by *Spodoptera frugiperda* in Brazil. *Pest Management Science* 72: 1727-1736.

Ranjith, M.T., A. Prabhuraj, and Y.B. Srinivas. 2010. Survival and reproduction of natural populations of *Helicoverpa armigera* on Bt-cotton hybrids in Raichur, India. *Current Science* 99: 1602-1606.

Ravi, K.C., K.S. Mohan, T.M. Manjunath, G. Head, B.V. Patil, D.P. Angeline Greba, K. Premalatha, J. Peter, and N.G.V. Rao. 2005. Relative abundance of *Helicoverpa armigera* (Lepidoptera: Noctuidae) on different host crops in India and the role of these crops as natural refuge for *Bacillus thuringiensis* cotton. *Environmental Entomology* 34: 59-69.

Singla, R., P. Johnson, and S. Misra. 2012. Examination of regional-level efficient refuge requirements for Bt cotton in India. *AgBioForum* 15: 303-314.

Specht, A., D.R. Sosa-Gómez, S.V.D. Paula-Moraes, and S.A.C. Yano. 2013. Morphological and molecular identification of *Helicoverpa armigera* (Lepidoptera: Noctuidae) and expansion of its occurrence record in Brazil. *Pesquisa Agropecuária Brasileira* 48: 689-692.

Storer, N.P., M.E. Kubiszak, J.E. King, G.D. Thompson, and A.C. Santos. 2012. Status of resistance to Bt maize in *Spodoptera frugiperda*: lessons from Puerto Rico. *Journal of Invertebrate Pathology* 110: 294-300.

Tabashnik, B.E., and Y. Carrière. 2017. Surge in insect resistance to transgenic crops and prospects for sustainability. *Nature Biotechnology* 35: 926-935.

Tabashnik, B.E., A.J. Gassmann, D.W. Crowder, and Y. Carrière. 2008. Insect resistance to Bt crops: Evidence versus theory. *Nature Biotechnology* 26: 199-202.

Tabashnik, B. E., J. B. J. Van Rensburg, and Y. Carrière. 2009. Field-Evolved Insect Resistance to Bt Crops: Definition, Theory, and Data. *Journal of Economic Entomology* 102: 2011-2025.

Tabashnik, B.E., T. Brévault, and Y. Carrière. 2013. Insect resistance to Bt crops: lessons from the first billion acres. *Nature Biotechnology* 31: 510-521.

U.S. Environmental Protection Agency. 1998. The Environmental Protection Agency's White Paper on Bt Plant-Pesticide Resistance Management. Environmental Protection Agency, EPA Publication 739-S-98-001, Washington, DC.

U.S. Environmental Protection Agency. 2001. Biopesticides registration action document: *Bacillus thuringiensis* plant-incorporated protectants. Biopesticides and Pollution Prevention Division, Washington, DC. https://www3.epa.gov/pesticides/chem_search/reg_actions/pip/bt_brad.htm

U.S. Environmental Protection Agency. 2018. Framework to delay corn rootworm resistance. https://www.epa.gov/regulation-biotechnology-under-tsca-and-fifra/framework-delay-corn-rootworm-resistance.

Walker, K., M. Mendelsohn, S. Matten, M. Alphin, and D. Ave 2003. The role of microbial Bt products in US crop protection. *Journal of New Seeds* 5: 31-51.

Welch, K.L., G.C. Unnithan, B.A. Degain, J. Wei, J. Zhang, X. Li, B.E. Tabashnik, and Y. Carrière 2015. Cross-resistance between toxins in pyramided Bt crops and resistance to Bt sprays in *Helicoverpa zea*. *Journal of Invertebrate Pathology* 132: 149-156.

Wilson, L., S.J. Downes, G. Baker, M. Khan, M. Whitehouse, and S. Mass. 2013. IPM in the transgenic era: A review of challenges from emerging pests. *Crop and Pasture Science* 64: 737–749.
